# Delivering mental health and psychosocial support interventions to women and children in conflict settings: a systematic review

**DOI:** 10.1136/bmjgh-2019-002014

**Published:** 2020-03-15

**Authors:** Mahdis Kamali, Mariella Munyuzangabo, Fahad J Siddiqui, Michelle F Gaffey, Sarah Meteke, Daina Als, Reena P Jain, Amruta Radhakrishnan, Shailja Shah, Anushka Ataullahjan, Zulfiqar A Bhutta

**Affiliations:** 1 Centre for Global Child Health, Hospital for Sick Children, Toronto, Ontario, Canada; 2 Health Services and Systems Research, Duke-NUS Graduate Medical School, Singapore, Singapore; 3 Center of Excellence in Women and Child Health, Aga Khan University, Karachi, Pakistan

**Keywords:** child health, health services research, mental health & psychiatry, public health, systematic review

## Abstract

**Background:**

Over 240 million children live in countries affected by conflict or fragility, and such settings are known to be linked to increased psychological distress and risk of mental disorders. While guidelines are in place, high-quality evidence to inform mental health and psychosocial support (MHPSS) interventions in conflict settings is lacking. This systematic review aimed to synthesise existing information on the delivery, coverage and effectiveness of MHPSS for conflict-affected women and children in low-income and middle-income countries (LMICs).

**Methods:**

We searched Medline, Embase, Cumulative Index of Nursing and Allied Health Literature (CINAHL) and Psychological Information Database (PsycINFO)databases for indexed literature published from January 1990 to March 2018. Grey literature was searched on the websites of 10 major humanitarian organisations. Eligible publications reported on an MHPSS intervention delivered to conflict-affected women or children in LMICs. We extracted and synthesised information on intervention delivery characteristics, including delivery site and personnel involved, as well as delivery barriers and facilitators, and we tabulated reported intervention coverage and effectiveness data.

**Results:**

The search yielded 37 854 unique records, of which 157 were included in the review. Most publications were situated in Sub-Saharan Africa (n=65) and Middle East and North Africa (n=36), and many reported on observational research studies (n=57) or were non-research reports (n=53). Almost half described MHPSS interventions targeted at children and adolescents (n=68). Psychosocial support was the most frequently reported intervention delivered, followed by training interventions and screening for referral or treatment. Only 19 publications reported on MHPSS intervention coverage or effectiveness.

**Discussion:**

Despite the growing literature, more efforts are needed to further establish and better document MHPSS intervention research and practice in conflict settings. Multisectoral collaboration and better use of existing social support networks are encouraged to increase reach and sustainability of MHPSS interventions.

**PROSPERO registration number:**

CRD42019125221.

**Key questions**

**What is already known about this topic?**

- Armed conflicts are linked to mental health consequences that disproportionately burden vulnerable populations such as women and children, though empirical evidence on the mechanisms of intervention implementation and delivery is lacking.

**What are the new findings?**

- The majority of interventions delivered to children and adolescents were delivered in specialised centres and schools.
- School-based interventions appear to be effective at improving mental health outcomes in children and adolescents.
- Interventions targeted to women exclusively are very limited.
- Access and security to target populations, language and culture and inadequate infrastructure are some reported barriers to delivering mental health interventions.

**Recommendations for policy**

- Along with strengthening the documentation of research and practice of intervention delivery in conflict settings, more concerted efforts are needed to incorporate these interventions into established networks and resources.

## Background

Globally, 172 million people are affected by armed conflict, and at least 240 million children live in countries affected by conflict or fragility.^1 2^ Humanitarian crises, such as armed conflicts, are linked to increased levels of psychological distress and higher risk of an array of mental disorders.^3–5^ Complexities further increase when unstable situations result in large-scale displacement and migration, exposing populations to stressful events prior to departure, during transit and after arrival, as they cope with integrating in a new environment or country.^6^ The mental health consequences of armed conflict are disproportionately experienced by children, who are already undergoing rapid and complex physiological, cognitive and emotional changes.^7^ Exposure to war, conflict and terror interferes with children’s natural development and has shown to lead to elevated levels of post-traumatic stress, anxiety, depression and many behavioural and emotional reactions.^7 8^

Empirical evidence on the delivery of effective mental health and psychosocial support (MHPSS) interventions to the most vulnerable populations, women and children, in humanitarian settings is scant. The 2017 Lancet series on health in humanitarian crises confirmed the dire necessity for vigorous, high quality and practical evidence to help inform interventions in these settings.^9^ The scarcity of empirical evidence is coupled with a lack of consensus on terminology, measurement and monitoring and evaluation of MHPSS interventions.^10^ As an attempt to safeguard best practices for MHPSS in emergency settings, WHO initiated the Inter-Agency Committee Guidelines for MHPSS in emergency settings in 2007.^11^ They provide standards on implementing minimum, yet essential, responses as quickly as possible during emergencies. Additionally, the Sphere Handbook has become a reference tool on universal minimum standards for humanitarian response, but a particular focus on MHPSS is lacking.^12^

Previous literature reviews and evidence syntheses have been conducted on the prevalence of mental health disorders among conflict-affected persons,^5 8 13–22^ but these were limited to certain mental health conditions, focused on high-income settings, or are not up-to-date. Stronger scientific evidence on implementing effective MHPSS interventions is needed in order to adequately and effectively respond to humanitarian crises. This current systematic review aims to help fill this gap by synthesising existing data and information on how MHPSS interventions are being implemented and delivered in conflict settings, and examining the reported coverage and observed effects of these interventions on mental health outcomes.

## Methods

### Indexed literature search

We conducted a systematic search of literature published between January 1 1990 and 31 March 2018 in Medline, Embase, CINAHL and PsycINFO using search terms related to three concepts: (1) conflict; (2) women and children; and (3) MHPSS. Conflict-related terms included “war”, “crisis”, “refugees”, “internally displaced person (IDP)” and “stateless”. Population-related words included “women”, “children”, “pregnant”, “adolescents”, and “newborn”. MHPSS-related terms included “mental disorders”, “mental illness”, “mental symptoms”, “psychiatric diagnoses”, and so on. The complete Medline search syntax is included in online supplementary appendix A. We also screened the reference lists of relevant systematic reviews conducted previously.

10.1136/bmjgh-2019-002014.supp1 Supplementary data

Grey literature was searched through the websites of 10 major humanitarian agencies and organisations who are actively involved in responding to or researching conflict situations: Emergency Nutrition Network, International Committee of the Red Cross, International Rescue Committee, Médecins Sans Frontières, Save the Children, United Nations Population Fund, United Nations High Commissioner for Refugees, UNICEF, Women’s Refugee Commission and World Vision. We used broad terms for MHPSS interventions in conflict-affected populations, tailored to the search functionality of each website. Due to the large volume of grey literature available, we reviewed grey literature documents published from 1 January 2013 to 30 November 2018 only, for feasibility.

### Eligibility criteria

Eligible publications were limited to those reporting on conflict-affected populations in low-income or middle-income countries, as classified by the World Bank.^23^ The publication must have described an MHPSS intervention being delivered during or within 5 years of cessation of a conflict. The intervention had to target or include neonates, children, adolescents or women of reproductive age. In order to identify the most informative resources from the large volume of grey literature available, eligible grey literature publications also had to report on both the delivery site and delivery personnel for each intervention.

Non-English publications; case reports of single patients; or publications reporting on military personnel, refugee populations bound for high-income countries, surgical techniques, economic or mathematical modelling were excluded. Other excluded publications included systematic reviews, guidelines and studies where no specific health intervention was described.

### Data extraction and analysis

All identified indexed records were downloaded into EndNote software and duplicates were removed. Unique records were then imported into Covidence software for screening. Titles and abstracts were reviewed in duplicate and the full-text of potentially relevant publications studies was screened for eligibility by a single reviewer.

We extracted data and information from all indexed and grey literature publications that met the eligibility criteria in duplicate, using a customised form in REDCap software. Key variables with regard to setting, target population, study design and intervention descriptions were extracted. The double-entered data were compared and any inconsistencies were resolved through discussion or by a third reviewer if needed.

Descriptive statistics were used to summarise key characteristics of the conflict and intervention, including displacement status and delivery characteristics, and we narratively synthesised information on the factors impeding or facilitating intervention delivery. We tabulated reported intervention coverage and effectiveness data. Due to the substantial methodological heterogeneity among publications, meta-analyses were not attempted.

## Results

**Figure 1:**
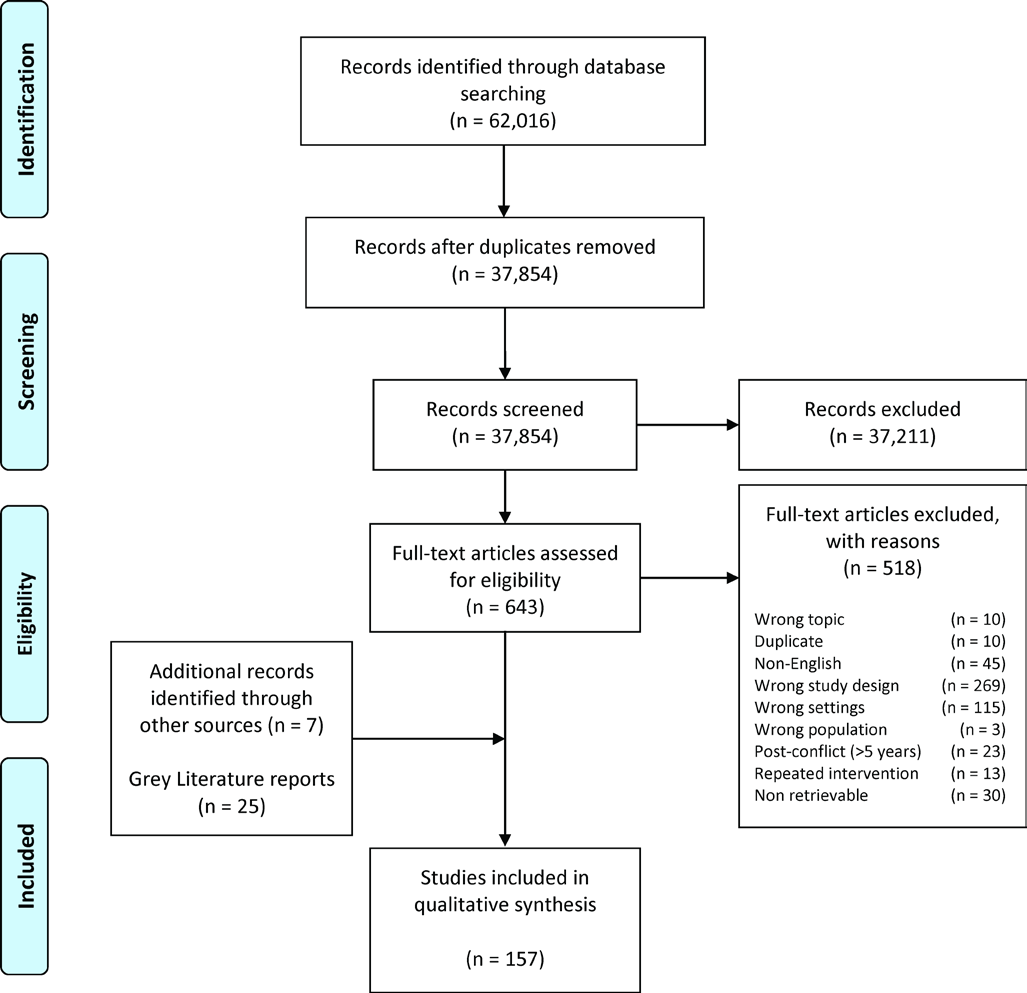
Preferred Reporting Items for Systematic Reviews and Meta-Analyses (PRISMA) flow diagram of literature selection.

The initial electronic search yielded 37 854 unique records, with most being excluded after title and abstract screening as they did not meet the review eligibility criteria (figure 1). Of the 643 potentially relevant publications screened in full text, 125 were identified as eligible for this review. The grey literature search identified another 25 eligible publications, and a further seven were identified through reference list of previous reviews, with a total of 157 publications finally included in the review.^24–154^Table 1 summarises the characteristics of all included publications. Further details are presented in the online supplementary appendix B.

**Table 1:** Summary of characteristics of included studies (n=157)

Geographic region *†	n
Sub-Saharan Africa	65
Middle East and North Africa	36
Europe and Central Asia	26
South Asia	20
East Asia and Pacific	10
Latin America and the Caribbean	2
**Publication type**	**n**
Observational study	57
Non-research report	53
Randomised controlled trial	29
Non-randomised controlled trial	10
Qualitative study	7
Mixed methods	1
**Target population type†**	**n**
All/general population	77
Adolescents (10–19 years)	59
Children (13 months–9 years)	39
All women	26
**Displacement status of beneficiary population†**	**n**
Refugees	63
IDPs	56
Unreported	47
Non-displaced	16
Host	5
**Setting of displaced populations**	**n**
Unreported	69
Camp	45
Mixed	22
Dispersed	21

*World Bank Regions.

†Individual publications may contribute to multiple categories.

IDP, internally displace person.

The majority of publications reported on observational studies (n=57, 36%) or randomised controlled trials (n=29, 18%), or were non-research reports (n=53, 34%). Almost half, 43%, of the included publications (n=68) described interventions targeted at children and adolescents, while only 17% were targeted at women of reproduction age (n=26). Only six publications in the discussed interventions that were delivered to children and their caregivers simultaneously. The most common displacement status reported on was refugee populations and IDPs, and camps were the most prevalent setting of displaced populations.

A variety of mental health conditions was reported, ranging from post-traumatic stress disorder and trauma-related disorders to general mental health and functioning, with only one publication focusing on alcohol and substance abuse (online supplementary appendix B). Almost half of the publications, 44%, reported interventions being delivered solely through non-governmental organisation (NGO) platforms, and only 10% of publications reported interventions being delivered through NGO as well as education platforms.

**Figure 2:**
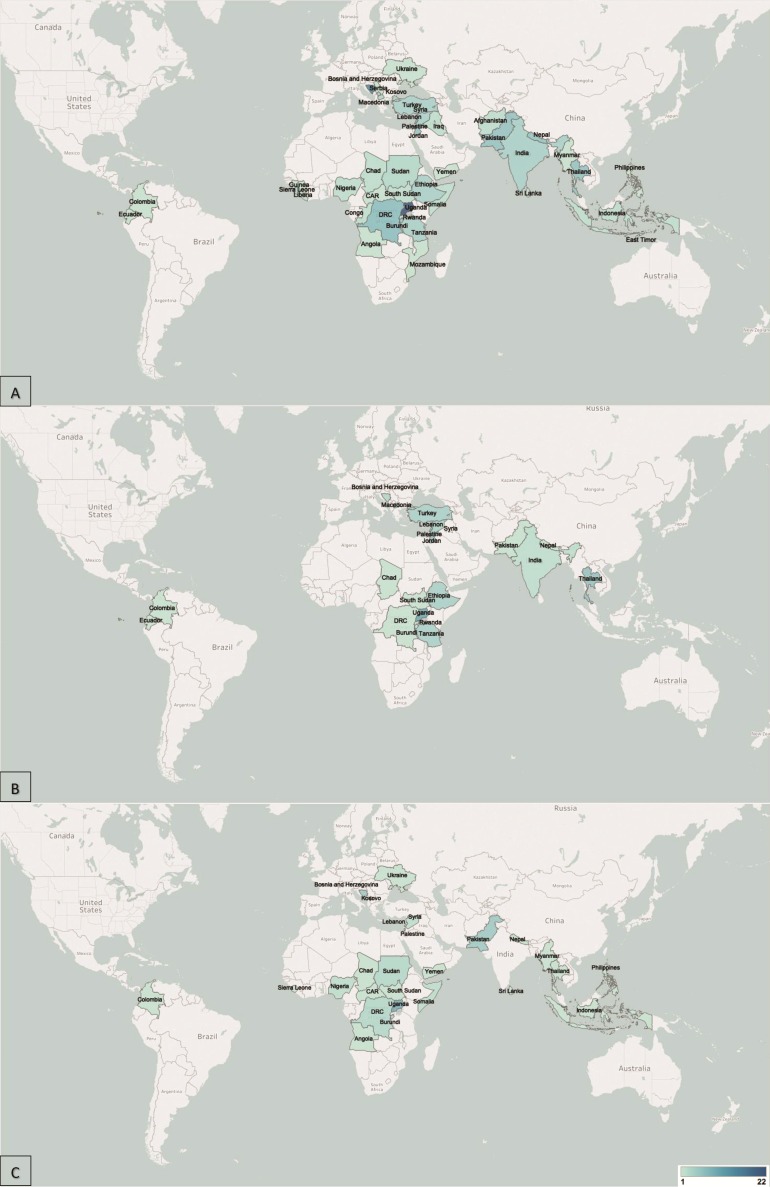
(A) Geographical distribution of included publications. (B) Geographical distribution of included publications, by refugee population displacement status. (C) Geographical distribution of included publications, by internally displaced population status.

The included publications were geographically diverse (figure 2A). The majority reported on conflict-affected populations in Sub-Saharan Africa, Europe and South Asia, with the highest concentration of publications coming from Uganda (22 studies). Latin America was the least represented region, with only Colombia and Ecuador being represented in one publication each. Stratified by the displacement status of the populations, the geographical distributions of publications reporting on refugees and IDPs followed broadly similar patterns, but no publications focused on refugees in West Africa, and more countries featured in reports of IDPs than of refugees, especially in Sub-Saharan Africa and South-East Asia (figure 2B and C).

**Figure 3:**
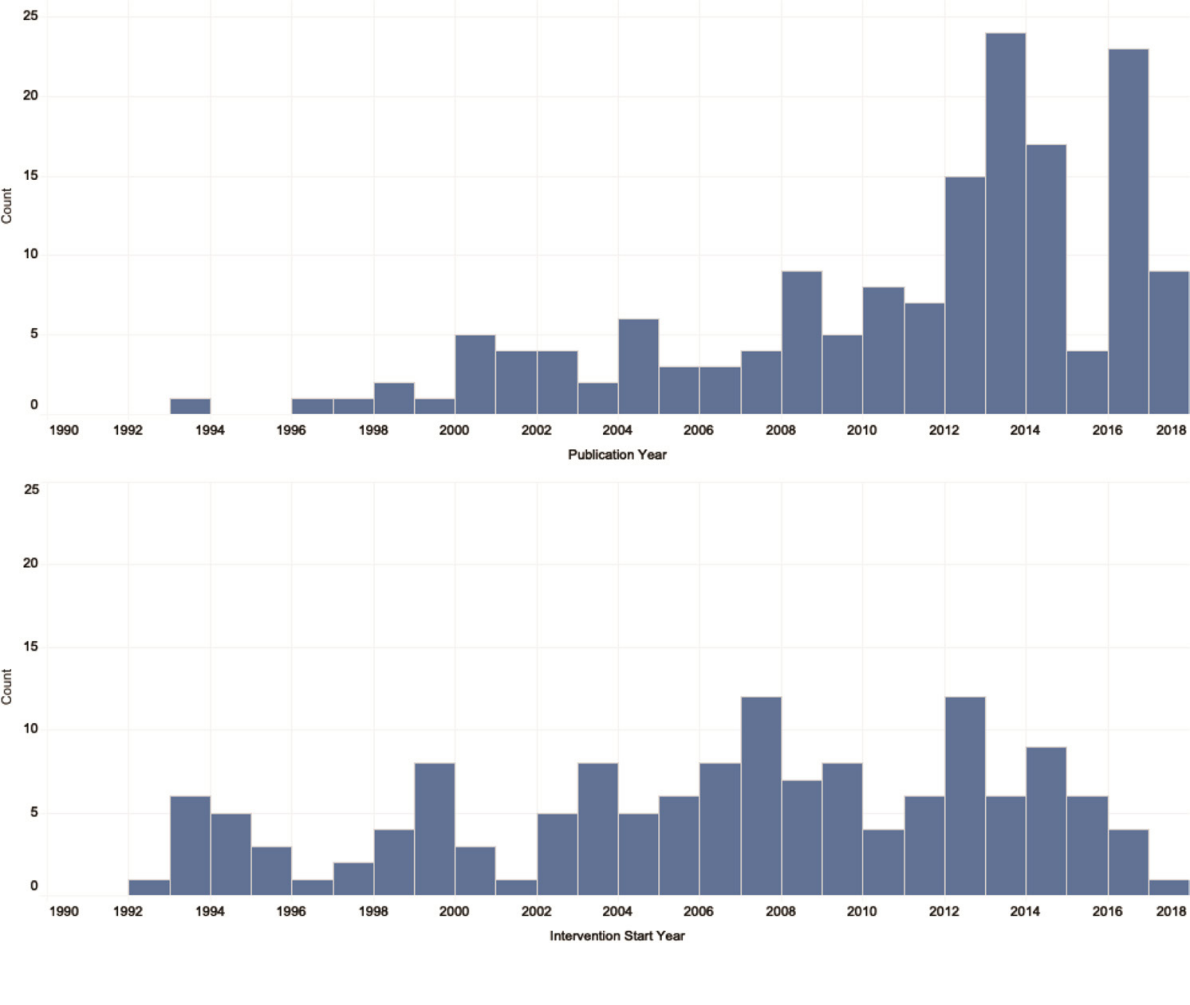
Publication counts by publication year and intervention start year.

Most of the literature on the delivery of MHPSS interventions for women and children in conflict settings is recent, with the majority published after 2012, and with peaks in 2013 and 2016 (figure 3). When examining the years that reported interventions was first delivered, the included publications are more widely dispersed over time, with peaks in 2007 and 2012.

**Figure 4:**
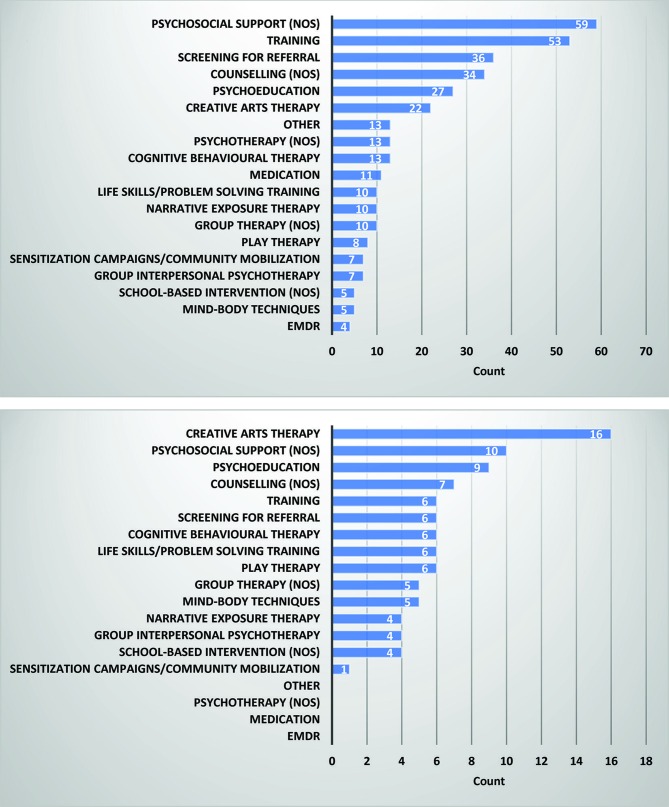
Reporting frequency of mental health and psychosocial support interventions for all ages (top) and for children aged 5–19 (bottom). EMDR, eye movement desensitisation and reprocessing; NOS, not otherwise specified.

Psychosocial support was the most frequently reported intervention delivered to all study populations, followed by training interventions and then by screening (for referral/with intention to treat) (figure 4A). The delivery of counselling, creative arts therapy and psychoeducation interventions were also reported relatively frequently. Several types of therapy including eye movement desensitisation and reprocessing (EMDR), mind–body techniques and group interpersonal psychotherapy were reported with similar, low frequency. Interventions targeted at children and adolescents followed a different pattern, where the delivery of creative arts therapy was reported most frequently, followed by psychosocial support (figure 4B).

When interventions were mapped to the places and levels of care at which they were delivered, different patterns are evident for those interventions delivered to all populations (n=345) compared with those targeted at children and adolescents (n=95; table 2).

**Table 2:** Delivery characteristics of interventions, by age group (all ages, and 5–19 years) and site of delivery

Interventions for all ages (n=345)	Outpatient				Outreach		Community based			
	Hospitals	Clinics	Research centres	Specialised centres	Web based	Health posts/mobile clinics	Home	Place of worship	Schools	Community centres
Counselling										
Screening for referral
Counselling (NOS)
Cognitive–behavioural therapy
Creative arts therapy
EMDR
Group therapy										
Group interpersonal psychotherapy
Group therapy (NOS)
Psychotherapy										
Narrative exposure therapy
Play therapy
Psychoeducation
Psychosocial support (NOS)
Psychotherapy (NOS)
Technique/skills										
Mind–body techniques
Life skills/problem-solving training
School-based intervention (NOS)
Other interventions										
Medication
Sensitisation campaigns/community mobilisation
Other
Training

NOS, Not Otherwise Specified.

**Table:** 

	Outpatient				Outreach		Community based			
Interventions for children 5–19 years (n=95)	Hospitals	Clinics	Research centres	Specialised centres	Web based	Health posts/mobile clinics	Home	Place of worship	Schools	Community centres
Counselling										
Screening for referral
Counselling (NOS)
Cognitive–behavioural therapy
Creative arts therapy
EMDR
Group therapy										
Group interpersonal psychotherapy
Group therapy (NOS)
Psychotherapy										
Narrative exposure therapy
Play therapy
Psychoeducation
Psychosocial support (NOS)
Psychotherapy (NOS)
Technique/skills										
Mind–body techniques
Life skills/problem-solving training
School-based intervention (NOS)
Other interventions										
Medication
Sensitisation campaigns/community mobilisation
Other
Training

EMDR, eye movement desensitisation and reprocessing; NOS, Not otherwise specified.

Among interventions delivered to populations of all ages, psychosocial support and screening for referral were delivered broadly across all sites and at different levels of care, while EMDR was only delivered in health posts/mobile clinics. Interventions targeted to children and adolescents, however, were almost exclusively delivered in specialised centres and schools, with only counselling and psychosocial support being delivered more widely. Only two interventions were delivered to children and adolescents through an outreach approach, with screening being delivered through a web-based modality, and counselling being delivered at health posts or mobile clinics. With the exception of schools, community-based delivery approaches were lacking for interventions targeted to youth, and no interventions were delivered at the home.

Different patterns for children and adolescents compared with the general population are also evident when interventions were mapped to the personnel delivering them (table 3).

**Table 3:** Delivery characteristics of interventions, by age group (all ages, and 5–19 years) and personnel

Interventions for all ages (n=345)	Physicians/ psychiatrists	Counsellors/ therapists/ psychologists	Other mental health professionals	Nurses	Health workers	Social workers	NGO staff/researchers	Facilitators/ trainers	CHWs	Trained civilians/ volunteers	Teachers	Civic leader/ religious leaders
Counselling												
Screening for referral
Counselling (NOS)
Cognitive–behavioural therapy
Creative arts therapy
EMDR
Group therapy												
Group interpersonal psychotherapy
Group therapy (NOS)
Psychotherapy												
Narrative exposure therapy
Play therapy
Psychoeducation
Psychosocial support (NOS)
Psychotherapy (NOS)
Technique/skills												
Mind–body techniques
Life skills/problem-solving training
School-based intervention (NOS)
Other interventions												
Medication
Sensitisation campaigns/community mobilisation
Other
Training

CHWs, Community Health Workers; NGO, Non-governmental organization; NOS, Not otherwise specified.

**Table:** 

Interventions for children 5–19 years (n=95)	Physicians/ Psychiatrists	Counsellors/ Therapists/ Psychologists	Other mental health professionals	Nurses	Health workers	Social workers	NGO staff/ Researchers	Facilitators/ Trainers	CHWs	Trained civilians/ Volunteers	Teachers	Civic leader/ Religious leaders
Counselling												
Screening for referral						
Counselling (NOS)										
Cognitive behavioural therapy						
Creative arts therapy						
EMDR												
Group Therapy												
Group interpersonal psychotherapy										
Group therapy (NOS)						
Psychotherapy												
Narrative exposure therapy							
Play therapy								
Psychoeducation								
Psychosocial support (NOS)						
Psychotherapy (NOS)												
Techniques/Skills												
Mind-body techniques								
Life skills/Problem solving training								
School-based intervention (NOS)									
Other Interventions												
Medication												
Sensitisation campaigns /community mobilisation										
Other												
Training										

EMDR, eye movement desensitisation and reprocessing; NGO, non-governmental organisation; NOS, not otherwise specified.

Psychiatrists and physicians delivered a wide range of interventions in the general population, but only screening for referral and group therapy was delivered by these professionals to youth specifically. NGO staff/researchers and counsellors/therapists/psychologists delivered the widest range of interventions in both populations. Nurses and community health workers did not deliver any interventions to children and adolescents, and civic/religious leaders only delivered psychosocial support. In the general population, psychosocial support was delivered by all types of personnel, with psychoeducation and cognitive–behavioural therapy (CBT) also delivered by many types. Similarly, in the 5–19 year age group, CBT was delivered by the widest range of personnel, followed by screening for referral and psychosocial support.

Relatively few publications that reported on the delivery of MHPSS interventions to women and children in conflict settings also reported on intervention coverage or effectiveness. Coverage data were available from only two publications included in our review,^96 99^ focusing on counselling and EMDR in adolescents and women, respectively. Quantitative data relating to the effect of interventions were available from 17 publications.^29 33 41 55 57 67 68 84 98 106 113 141 144 146 147 149 150^ Reported outcomes included anxiety-related symptoms, depression-related symptoms, general mental health and functioning, post-traumatic stress disorder and trauma-related disorder. Nearly all interventions for which effectiveness data were reported were targeted to children and adolescents, with a majority delivered in schools and a large proportion delivered to IDPs. Most of the publications reporting quantitative data did not report final effect estimates, and the common non-reporting of measures of dispersion or correlation coefficients prevented our post-hoc calculation of such estimates. Furthermore, the heterogeneity of the settings, interventions and reported outcomes barred us from further synthesising the retrieved data on effectiveness. However, overall, school-based interventions or interventions delivered in school settings appear to be effective in reducing anxiety-related symptoms, depression-related symptoms post-traumatic stress disorder and general mental health and functioning in school-aged children. All intervention coverage and effectiveness data extracted from included publications are presented in online supplementary appendix C.

A number of key barriers to and facilitators of MHPSS intervention delivery were identified from the reviewed literature and grouped into themes, with supporting excerpts from the publications presented in table 4. Barriers to the delivery of interventions included limited access to target populations as a result of security constraints, in addition to infrastructure impediments such as limited network coverage and bombing of clinics. Language and cultural barriers also existed where implementers had difficulty communicating with the target population, and vice versa. In order to manage this, some programme trained lay staff from like communities; this strategy encountered other challenges however, where community members were dealing with their own trauma, or were lacking basic education, and were therefore unable to deliver services. Other barriers relating to the heterogeneity of study populations made it difficult to implement uniform interventions to wide groups of individuals. Many tactics to overcome the reported barriers were outlined in the literature. While training of lay staff encompassed some hindrances, generally, the utilisation of local community members was an important facilitating aspect of the delivery of MHPSS interventions, where doing so allowed for increased access to and acceptability among target populations. Training of outreach volunteers was also found to be imperative for being able to reach and refer people in need to the correct services and facilities. Another facilitator in the delivery of MHPSS interventions was integrating such interventions into existing networks and adapting them to the specific context. Such integration was especially helpful when they were incorporated into school-based programme, as they created safe environments for affected youth.

**Table 4:** Reported barriers to and facilitators of intervention delivery

Barriers	
Theme	Examples/excerpts
Access and security	“A continuous problem was the security situation, which impacted implementation of the programmes from the level of access to the target population, selection of staff and development of communication, to the identification of local support mechanisms”^132^ “There are always security risks to staff members due to the presence of landmines and unexploded ordinances, in addition to general travel restrictions imposed by peace-keeping troops that directly affect the sample selection process”^69^ “Time was another major constraint during these camps as, due to security concerns, travelling was not possible after dark”^76^ “Another obstacle was that the program coordinators were not allowed to travel to Iraq due to the worsened security situation. They were therefore forced to organize and supervise the program via the Internet, Skype, and by telephone”^152^
Infrastructure	“Poor network coverage and phone charging facilities in the settlement makes community mobilisation processes difficult for the implementation team”^103^ “The recent bombing and closure of the clinic resulted in a huge loss for the local communities’ leaving a gap in critical services for the health system of this war ravaged region”^73^ “In these settings, the context is likely to change abruptly at any moment, and the research subjects who are participating out of goodwill alone may no longer be available. The sudden change in political circumstances meant that the original plan to measure postintervention outcomes at 4 months after admission to the nutrition program had to be modified”^106^
Language and culture	“There was a huge language barrier as two-thirds of the patients could only communicate in Pashto. Even the Pashto speaking professionals had difficulty conducting interviews because of the dialect spoken by most IDPs”^76^ “Throughout the intervention programme, it was observed that the majority of the children who participated in the study could not name or explain their emotions”^149^
Training lay staff	“A limitation involves training teachers who had no mental health background and whose own mental state following the war trauma was a factor we could not control for completely (despite addressing it in their training)”^84^ “It was a challenge to find refugees resident in the settlement with even basic education. Many had had their schooling interrupted by the conflicts from which they fled, and most of those with some education had already left the settlement in search of employment opportunities”^117^
Heterogeneity of study population	“There was a broad age range, not all participants had been abducted and there was a broad range of reported psychopathology at pre-test assessment”^113^ “Other limitations were: not investigating the process of the intervention, not involving parents, the large size and developmental heterogeneity of the intervention groups, and the different treatment requirements for PTSD and depression”^143^

Facilitators	
Theme	Example/excerpts
Utilisation of local community members	“These psychosocial outreach volunteers represent the foundation of the mental health and psychosocial support programme. This is, in part, due to the fact that Syrian and refugee outreach volunteers are able to access areas that UNHCR staff are currently not permitted to visit, including collective shelters and communities hosting displaced Syrians. The outreach volunteers and the mobile MHPSS case managers are therefore able to provide a crucial outreach function by helping to decentralise psychosocial support and to bring mental health services closer to vulnerable populations”^70^ “The use of local volunteers and program graduates promotes community as well as enables the program more flexibility to reach out to a wider range of war-affected children in Kosovo”^66^ “The intervention which can be easily delivered by trained community volunteers (CVs). The CVs in our study by virtue of residing in these camps had the advantage of sharing the same language and culture of the refugees, thereby enhancing their acceptability”^151^
Training of outreach volunteers for referrals	“Community outreach in DRC was also an important source of referral. This outreach by MSF includes specific information on sexual violence services, which may be a reason for the high proportion of survivors of sexual violence, including children, presenting to the programme”^96^ “Community-based activities allowed outreach to a wide range of people among those in need as well as those who were not in need. After defining the scope of the intervention, the coverage of the refugee population could be extended to districts other than the ones previously identified, according to the population needs”^91^
Integration of MHPPS interventions into existing PHC/referral networks	“The main difference between the two projects resided in the network; in a center-based program, the creation of the referral system toward the center relied mostly on the pre-existing network of service providers (NGOs, community-based organizations, local institutions), while a community-based program created its own network, usually in collaboration with community leaders and key persons such as shopkeepers”^91^ “In DRC, the integrated nature of the programme and links with community health workers is reflected in the fact that almost half of all patients were referred by primary healthcare services, whereas in Iraq the focus on links to secondary and tertiary health services meant they were of more importance than primary care providers. Specific provision of information on child mental health services, by providers of those services, to groups that come into contact with children appears to improve uptake”^96^
Adaptation of interventions	“Using therapeutic consultations, mainly at the patient’s residence and often involving the family, more than three-quarters of patients had improved at the end of therapy. The importance of this adapted therapy, based on the psychodynamic approach and respecting the Palestinians’cultural characteristics, was possible and efficient”^65^ “In the absence of trained professionals for emergency interventions, mobilizing teachers was the best alternative to reach the largest number of students”^84^
School-based approach	“School-based intervention is the best way to reach a large number of children suffering from conflict-related distress and to target youth in their natural school environment by training professionals in the educational system. School-based programs allow youth to be trained and treated in a non-stigmatizing environment with little disruption to their daily schedules, which is important for mitigating posttraumatic stress“^24^ “Underscoring program sustainability, the Federal Ministry incorporated implementing the program into the counselors’ job requirements and contracted local mental health professionals to provide supervision. The resulting professional network interlinked participating schools with local mental health clinics and the local university and allowed the program to run autonomously throughout the school year“^90^

DRC, Democratic Republic of Congo; IDP, internally displaced person; MHPSS, mental health and psychosocial support; MSF, Médecins Sans Frontières; NGO, non-governmental organisation; PHC, primary health centre; PTSD, post-traumatic stress disorder; UNHCR, United Nations High Commissioner for Refugees.

## Discussion

This review provides a comprehensive overview of reported strategies and approaches used to deliver MHPSS interventions to conflict-affected women and children in low-income and middle-income countries, drawing on 157 indexed and grey literature publications identified using rigorous systematic review methods. Evidence syntheses in the past have examined the prevalence of mental health disorders in conflict-affected populations^5 8 13–19^; however, this review is the first, to our knowledge, to assess the mechanisms of intervention delivery and implementation in the particular context of women and children impacted by conflict.

While the body of literature on MHPSS in humanitarian contexts is growing, much work remains to improve the breadth of evidence on intervention effectiveness as well as effective delivery strategies to increase the coverage of interventions. The majority of the publications included in the present review reported on observational rather than experimental research, and those research studies that employed experimental designs were typically conducted in stable settings, such as well-established refugee camps, with limited generalisability to other conflict settings.

Psychosocial support, training, screening (for referral/with intent to treat) and counselling accounted for more than half of the delivered interventions reported in the included publications. Only one publication was identified that focused on interventions related to alcohol or substance abuse disorders.^87^ Understanding that conflict-affected populations are at a higher risk of experiencing alcohol and drug misuse as a result of coping mechanisms,^19 155^ it is surprisingly to observe an absence of strategies to prevent substance abuse.

While more than 40% of included publications reported on interventions targeted to children and adolescents, very few reported on interventions engaging parents/caregivers along with their children. Considering the significant toll that conflict and displacement take on children and families by destroying pre-existing community structures and social networks, it is important to use parents and caregivers as natural support systems for children, rather than relying on external aid such as counsellors and teachers.^156^ Similarly, very few studies described interventions that were promoted or delivered by religious leaders or through faith-based delivery channels. Populations affected by crises tend to use faith as a coping mechanism and express a spiritual or religious identity in an effort to seek comfort.^12^ Inadequate use of these networks is a missed opportunity to increase the reach of MHPSS interventions. Additionally, with the exception of schools, other community-based delivery approaches were not widely used for interventions targeted to children and adolescents. Outreach and community-based platforms are particularly important in displacement settings as a means to strengthen and facilitate the empowerment of communities.^156^

Almost half of the reviewed literature describe local and international NGOs working independently of each other and government bodies. While some MHPSS interventions were delivered through existing health or education systems, sustainability and increased coverage would be achieved if NGO-run programmes could be delivered through formal health and education systems and leveraging existing resources and structures,^17 74^ considering humanitarian donor funds are generally short term. According to the WHO Mental Health Gap Action Programme guidelines, integration can done by training healthcare staff in the identification, management and appropriate referral of mental health cases.^157^ Such integration will ensure sustainability, increase accessibility, and reduce the associated stigma of mental health. Accessibility and reach of MHPSS interventions could be further amplified by packaging them with interventions in other sectors, such as nutrition. In 2006, WHO recommended integrating psychosocial stimulation into emergency feeding programme, for example, yet not many studies have evaluated the impact of such combined interventions in emergency settings.^18 158^ Multisectoral integration is an area that should be prioritised for MHPSS, as the most vulnerable populations tend suffer from comorbidities and might be more easily reached and more effectively helped by targeting these simultaneously.^106^ Moreover, this integration can help ensure programme sustainability and thereby reduce the unintended negative consequences of suspending or withdrawing MHPSS interventions that communities were previously able to access.

Additionally, given that language and culture are cited in the literature as key intervention delivery barriers, more evidence on the unintended consequences of delivering MHPSS interventions that are cultural insensitive is needed.^159^ Cultural sensitivity needs to be prioritised in the context of diagnosing mental health disorders in conflict settings, since the Diagnostic and Statistical Manual of Mental Disorders is developed for western settings.^160^ Cultural appropriateness can be achieved with high-quality training of implementors and mental health professionals specific to the context they are working in, and also through increased delivery of web-based or mobile app interventions where opportunities for face-to-face interactions are limited.^6^

A major limitation of the literature is the sizeable number of publications that do not report key information such as the site of intervention delivery or the personnel delivering the intervention, indicating the need for stronger scientific reporting. There was also much heterogeneity and non-standardisation in the reporting of intervention outcomes, making pooled analysis challenging. In addition to the limitations of the literature, several methodological limitations of this systematic review must also be noted. Due to capacity and feasibility, we limited our review to English-language publications only, and our search of the grey literature was comprehensive and systematic, but not exhaustive. We may therefore have missed other publications that may have contributed different information to this review.

## Conclusion

The growing literature, including among children, is encouraging, but more attention must be paid to establishing and documenting the research and practice of MHPSS delivery in a wider range of conflict contexts covering a wider range of interventions. Better leveraging of existing resources and structures in the health and the education sector, and others such as nutrition, could help improve the reach of MHPSS interventions and also their sustainability. So, too, could an increased focus on interventions that make better use of parents’/caregivers’ supportive roles in their children’s lives, as well as other pre-existing or emerging social support networks, and that rely less on the availability of specialised mental health personnel. More robust evaluation of intervention effectiveness as well as intervention delivery effectiveness would improve the evidence base, and could ultimately improve the mental health and psychosocial well-being of conflict-affected women and children globally.
