# Supplementary material for: Delivering mental health and psychosocial support interventions to women and children in conflict settings: a systematic review
Source: BMJ Glob Health. 2020 Mar 15;5(3):e002014. doi: 10.1136/bmjgh-2019-002014 (PMC7073823; doi:10.1136/bmjgh-2019-002014)
Supplement: Supplementary data [file bmjgh-2019-002014supp001.pdf]

Appendices

Appendix A: MEDLINE Search Strategy for the delivery of MHPSS interventions to women and children affected by conflict in LMICs 2

Appendix B: Characteristics of included publications 6

Appendix C: Reported quantitative data on intervention coverage and effectiveness, by outcome 20

Kamali M, *et al. BMJ Global Health* 2020; 5:e002014. doi: 10.1136/bmjgh-2019-002014

Appendix A: MEDLINE Search Strategy for the delivery of MHPSS interventions to women and children affected by conflict in LMICs

I. Conflict-related terms

1. disasters/ or emergencies/ or mass casualty incidents/
2. disaster victims/
3. ((disaster or disasters or catastrophe or catastrophes) adj5 (environ* or human or manmade or "man made" or nature or natural or weather)).tw,kf.
4. ("mass casualty" or "mass casualties" or "mass fatalities" or "mass fatality").tw,kf.
5. ((crisis or crises) adj5 (environ* or human or manmade or "man made" or nature or natural or weather)).tw,kf.
6. "warfare and armed conflicts"/ or armed conflicts/ or warfare/ or biological warfare/ or bioterrorism/ or chemical warfare/ or chemical terrorism/ or nuclear warfare/ or psychological warfare/ or war crimes/ or ethnic cleansing/ or genocide/ or holocaust/ or war exposure/ or war-related injuries/
7. afghan campaign 2001-/ or gulf war/ or iraq war, 2003-2011/
8. ("afghan campaign" or "armed conflict" or "armed conflicts" or "gulf war" or "iraq war" or "war time" or "wartime").tw,kf.
9. ((armed or zone or political or civil) adj3 (conflict or conflicts or attack or attacks or war or wars or "no fly")).tw,kf.
10. ("war related injuries" or "war related traumas" or "war related injury" or "war related trauma").tw,kf.
11. ("militant group" or "militant groups" or "militant organization" or "militant organizations" or "militant organisation" or "militant organisations").tw,kf.
12. ("biological terrorism" or bioterrorism or biowarfare or "chemical terrorism" or "ethnic cleansing" or "ethnic cleansings" or "gas poisoning" or genocide or holocaust or holocausts or "nuclear terrorism" or "war exposure" or "war exposures").tw,kf.
13. Disaster Medicine/
14. disease outbreaks/
15. Emergency Medical Services/
16. ((emergency or emergencies) adj5 (environ* or human or manmade or "man made" or nature or natural or weather)).tw,kf.
17. Starvation/
18. (famine or famines or starvation or starvations).tw,kf.
19. cyclonic storms/ or droughts/ or floods/ or tornadoes/ or tidal waves/
20. avalanches/ or earthquakes/ or landslides/ or tidal waves/ or tsunamis/ or volcanic eruptions/
21. (avalanche or avalanches or cyclone or cyclones or drought or droughts or earthquake or earthquakes or flood or flooded or flooding or floods or hurricane or hurricanes or landslide or landslides or "land slide" or "land slides" or mudslide or mudslides or "mud slide" or "mud slides" or storm or storms or tornado or tornadoes or tsunami or tsunamis or typhoon or typhoons or "volcanic ash" or "volcanic eruption" or "volcanic eruptions" or "volcanic gases").tw,kf.
22. refugees/
23. (evacuee or evacuees or refugee or refugees or squatter or squatters or transients).tw,kf.
24. relief work/ or rescue work/
25. ((rescue or relief or aid) adj (plan or plans or activity or activities or agency or agencies)).tw,kf.
26. ("aid plan" or "aid work" or "relief plan" or "relief work" or "rescue plan" or "rescue work").tw,kf.
27. ((staff or staffs or worker or workers) adj3 (relief or aid)).tw,kf.
28. (humanitarian assistance or humanitarian assistances or relief work or relief works).tw,kf.
29. (humanitarian adj2 (aid or response or relief or crisis or crises or emergency or emergencies or disaster or disasters)).tw,kf.
30. Altruism/

31. (humanitarianism or altruism).tw,kf.
32. ("displaced children" or "displaced families" or "displaced family" or "displaced individuals" or "displaced internally" or "displaced men" or "displaced people" or "displaced peoples" or "displaced person" or "displaced persons" or "displaced population" or "displaced populations" or "displaced women" or "forced displacement" or "forced displacements" or "internal displaced" or "internal displacement" or "internally displaced" or "population displaced" or "population displacement").tw,kf.
33. (((camp or camps) and displac*) or "protected village*").tw,kf.
34. (victim or victims).tw,kf.
35. rubble.tw,kf.
36. or/1-35

II. Population of interest

37. adolescent/ or young adult/
38. (adolescence or adolescent or adolescents or teen* or youth or youths or "young adult" or "young adults").tw,kf.
39. Pregnant Women/
40. exp pregnancy/
41. (expectant or expectancy or gravid* or pregnant or pregnancies or pregnancy).tw,kf.
42. ("mother to be" or "mothers to be").tw,kf.
43. (prenatal or "pre natal").mp.
44. (perinatal or "peri natal").mp.
45. ((trimester or trimesters) adj3 (first or second or mid or third or final or "1st" or "2nd" or "3rd")).tw,kf.
46. (midtrimester or midtrimesters or "early placental phase" or "early placental phases").tw,kf.
47. exp Delivery, Obstetric/
48. ((labor or labour) adj5 (birth* or breech or childbirth or childbirths or complicat* or difficult or early or easy or induce* or induction or late or obstetric* or onset or pregnan* or present*)).tw,kf.
49. parturients.tw,kf.
50. (birth or births or childbirth or childbirths or parturition or parturitions).tw,kf.
51. ("abdominal deliveries" or "abdominal delivery" or "c-section" or "c-sections" or caesarean or caesareans or cesarean or cesareans or "postcesarean section" or "postcaesarean section").tw,kf.
52. exp Abortion, Induced/
53. (abortion or abortions or embryotomies or embryotomy or "postconception fertility control").tw,kf.
54. ((pregnancy or pregnancies) adj3 terminat*).tw,kf.
55. "sexually active".tw,kf.
56. child/ or child, preschool/ or infant/ or infant, newborn/ or infant, low birth weight/ or infant, small for gestational age/ or infant, very low birth weight/ or infant, extremely low birth weight/ or infant, postmature/ or infant, premature/ or infant, extremely premature/
57. (infan* or newborn* or "new born*" or neonat* or baby* or babies or toddler* or boy or boys or boyfriend or boyhood or girl* or kid or kids or child* or pediatric* or paediatric* or peadiatric* or prematur* or preterm*).mp. or school*.tw.
58. refugees/
59. (refugee or refugees).tw,kf.
60. or/37-59
61. 36 and 60

III. Mental Health and Psychosocial Support

62. exp Mental Disorders/
63. ("mental change*" or "mental confusion" or "mental defect" or "mental defects" or "mental disorder" or "mental disorders" or "mental disturbance" or "mental disturbances" or "mental illness*" or "mental insufficienc*" or "mental symptom*" or "mentally ill" or "neurodevelopmental disorder*" or "neuropsychiatric

disease*" or "neuropsychiatric disorder*" or "psychiatric diagnosis" or "psychiatric disease*" or "psychiatric disorder*" or "psychiatric illness*" or "psychiatric symptom*" or "psychic disease*" or "psychic disorder*" or "psychic disturbance*" or "psychologic disorder*" or "psychologic disturbance*" or "psychological disorder*" or "psychological disturbance*").tw,kf.
64. ("alcohol abuse" or "alcohol addiction*" or "alcohol dependence" or "alcohol dependency" or "alcohol dependents" or "alcohol use disorder*" or "alcoholic" or "alcoholism" or "dipsomania" or "dipsomaniac" or "ethanol dependence" or "problem drinker*" or "problematic drinker*").tw,kf.
65. (alzheimer* or "diffuse cortical sclerosis").tw,kf.
66. (anxieties or anxiety or anxious* or hypervigilance or nervousness).tw,kf.
67. (delusion or delusions).tw,kf.
68. (dementia or dementias or amentia or amentias or demention).tw,kf.
69. (depression or depressions or "depressive episode*" or "depressive illness*" or "depressive state" or "depressive states" or "depressive symptom*" or "depressive syndrome*").tw,kf.
70. (aura or auras or epilepsia or epilepsies or epilepsy or epileptic or "falling sickness" or "seizure disorder" or "seizure disorders").tw,kf.
71. ((maladaptive or abnormal or dysfunctional) adj2 (grief or grieving or mourn*)).tw,kf.
72. (mania or manias or "manic behavior" or "manic behaviour" or "manic depressive psychoses" or "manic depressive psychosis" or "manic disorder*" or "manic episode*" or "manic state" or "manic states").tw,kf.
73. ("emotional trauma*" or "mental trauma*" or "psychic damage" or "psychic trauma*" or "psychical trauma*" or "psychologic trauma*" or "psychological trauma*").tw,kf.
74. (mood or moods).tw,kf.
75. (neuroses or neurosis or "neurotic disorder" or "neurotic disorders" or "neurotic onset" or "neurotic reaction*" or "neurotic syndrome*" or "neurotic tendency*" or "neuroticism" or "parapathia" or "psychoneuroses" or "psychoneurosis" or "psychoneurotic disease*" or "psychoneurotic disorder*" or "psychoneurotic disturbance*" or "psychoneurotic reaction*").tw,kf.
76. (ocd or "obsessive compulsive*" or "obsessional compulsive reaction*" or "obsessive syndrome*").tw,kf.
77. (panic or panics).tw,kf.
78. (phobia or phobias or phobic or claustrophobia* or claustrophobic).tw,kf.
79. (psychotic or psychoticism or psychoses or psychosis or schizoaffective or schizophreniform).tw,kf.
80. "psychological* health*".tw,kf.
81. psychosocial.tw,kf.
82. ("combat disorders" or "combat fatigue" or "combat stress" or "moral injuries" or "moral injury" or "post traumatic neuroses" or "post traumatic neurosis" or "posttraumatic neuroses" or "posttraumatic neurosis" or "posttraumatic psychic syndrome" or "posttraumatic stress" or "posttraumatic syndrome" or "ptsd" or "traumatic stress" or "traumatic stress disorder" or "war neurosis").tw,kf.
83. (schizophrenic or schizothymia or schizothyme).tw,kf.
84. Social Support/
85. ("social network" or "social networks" or "social support" or "social supports" or "community support" or "community supports" or "community network" or "community networks").tw,kf.
86. ("medically unexplained syndrome" or "medically unexplained syndromes" or "pain disorder" or "somatization disorder" or "somatization disorders" or "somatoform disorder" or "somatoform disorders").tw,kf.
87. (anguish or "emotional stress" or "emotional stresses" or "life stress" or "life stresses" or "psychologic stress" or "psychologic stresses" or "psychological stress" or "psychological stresses" or "stress reaction*" or "stress resistance*" or "stress response*" or "stress tolerance*" or suffering).tw,kf.
88. ("substance abuse*" or "abus* substance*" or "drug abuse" or "drug addiction*" or "drug use disorder*" or "substance addiction*" or "substance dependenc*" or "substance use disorder*" or "substance related disorder*").tw,kf.
89. (suicide or suicides or suicidal or "self killing").tw,kf.
90. (wellbeing or "well being").tw,kf.
91. or/62-90
92. 61 and 91

93. limit 92 to ed=20180401-20181231
94. ("2018 04*" or "2018 05*" or "2018 06*" or "2018 07*").dt.
95. 92 not (93 or 94) [exclude records added after Mar 31, 2018]

Kamali M, *et al. BMJ Global Health* 2020; 5:e002014. doi: 10.1136/bmjgh-2019-002014

Appendix B: Characteristics of included publications

Study ID	Report Type	Country	Duration of Intervention	Population	Displacement Type	Setting	Intervention(s)	Condition(s)	Delivery Characteristics		
									Platform	Site	Personnel
**Indexed Literature**											
Abdeen 2008	Observational Study	Palestine	2004-2005	Adolescents	Not reported	Not reported	Screening, training	PTSD	Education, research	Schools	Psychologists, research staff, teachers
Abramowitz 2005	Mixed Methods Study	Guinea	2003-2003	General	Not displaced	Not reported	Screening	GMHF	NGO	Not reported	NGO staff
Acarturk 2016	RCT	Turkey	2013-2014	General (excl. preg. women)	Refugees	Camp	EMDR	PTSD	Research	Camps	Psychologists, research staff
Acarturk 2015	RCT	Turkey	2013-2013	General (excl. preg, women)	Refugees	Camp	EMDR, psychoeducation	PTSD	Research	Camps	Psychologists, EMDR trainer, research staff
Ager 2011	RCT	Uganda	2007-2009	Children	IDPs	Camp, dispersed	Group therapy, creative arts therapy, training	GMHF	Education, NGO	Schools	NGO staff, teachers
Annan 2016	RCT	Thailand	2012-2013	Children, caregivers	Refugees	Urban & rural	Life skills/problem solving training, training	GMHF	NGO	Schools, community centres	CHWs, NGO staff, facilitators
Awodola 2012	Qualitative Study	Liberia	2006-2006	Children, adolescents	Not reported	Not reported	Psychoeducation, life skills/problem solving training, mind-body techniques, family reunification	GMHF	Healthcare, education, NGO	Schools, specialized centres	Counsellors, NGO staff
Ayoughi 2009	RCT	Afghanistan	2009-2010	Women	Not reported	Not reported	Counselling, medication	GMHF	Healthcare	Clinics	Counsellors, physicians
Badri 2012	Observational Study	Sudan	2010-2010	Women	IDPs, not displaced	Camp, rural	Counselling	GMHF	Education	Schools	Counsellors
Bass 2013	RCT	DRC	2010-2011	Women	Not reported	Not reported	Cognitive processing therapy, psychosocial support	Anxiety, Depression, PTSD	NGO	Research centres	Psychosocial assistants
Bass 2012	RCT	Indonesia	2007-2008	General	Not reported	Not reported	Counselling	GMHF	NGO	Not reported	Counsellors
Bastin 2013	Observational Study	Lebanon	2008-2011	Adults	IDPs, refugees	Camp	Psychosocial support, medication	GMHF	Healthcare, NGO	Clinics, hospitals	CHWs, social workers, nurses,

Kamali M, *et al. BMJ Global Health* 2020; 5:e002014. doi: 10.1136/bmjgh-2019-002014

Study ID	Report Type	Country	Duration of Intervention	Population	Displacement Type	Setting	Intervention(s)	Condition(s)	Delivery Characteristics		
									Platform	Site	Personnel
											psychiatrists, psychologists
Behrens 2012	Non-Research	Palestine	NR	Children	Not reported	Not reported	Creative arts therapy	Trauma-related disorder	Not reported	Not reported	Not reported
Benjamin 1996	Non-Research	Tanzania	1994-NR	Adolescents, women	Refugees	Camp	Counselling, creating support groups	Trauma-related disorder	NGO	Camps	Counsellors, social workers, volunteers
Betancourt 2010	Observational Study	Sierra Leone	2002-2004	Adolescents	Not reported	Not reported	Sensitization campaigns/community mobilization	GMHF	NGO	Specialized centres	NGO staff, health workers
Betancourt 2012	Observational Study	Ethiopia	2001-2002	Adolescents	Refugees	Camp	Psychosocial support, psychoeducation, play therapy	GMHF, internalizing/externalizing symptoms	Education, NGO	Schools	NGO staff
Betancourt 2013	Observational Study	Sierra Leone	2002-2008	Adolescents	Not reported	Not reported	Screening	PTSD	NGO	Specialized centres	NGO staff, researchers, social workers
Betancourt 2012	RCT	Uganda	NR	Adolescents	IDPs	Camp	Creative arts therapy, group interpersonal psychotherapy	Depression	NGO, research	Camps	NGO staff, researchers
Bhardwaj 2018	Observational Study	Nepal	2007-2012	Adolescents	Not reported	Not reported	Counselling	Suicidal thoughts	NGO	Not reported	Counsellors
Bolton 2014	RCT	Thailand	2011-2012	General	Refugees	Camp	Psychotherapy, training	Anxiety, Depression	NGO	Clinics, home	Counsellors, supervisors
Chase 2002	Non-Research	Sri Lanka	1993-1996	General	Not reported	Not reported	Psychosocial support, creative arts therapy, training	PTSD	Education, NGO	Schools	Counsellors, psychologists, therapists, teachers, trained local pop'n
Chauvin 1998	Non-Research	Rwanda	1995-NR	General	Not reported	Not reported	Sensitization campaigns/community mobilization, training, counselling	Trauma-related disorder	NGO	Clinics, schools, electronic/print	NGO staff

Study ID	Report Type	Country	Duration of Intervention	Population	Displacement Type	Setting	Intervention(s)	Condition(s)	Delivery Characteristics		
									Platform	Site	Personnel
Claessens 2012	Non-Research	Uganda	2009-2009	Adolescents	Not reported	Not reported	Life skills/problem solving training, training	GMHF	NGO	Not reported	Social workers
Cliff 1993	Non-Research	Mozambique	1993-NR	Children	Not reported	Not reported	School-based intervention, training	GMHF	Education	Schools	Teachers
Cohen 2016	Qualitative Study	Thailand	2014-2014	General	Refugees	Camp, rural	Counselling	GMHF	NGO	Clinics	NGO staff
Cohen 2013	Non-Research	Ecuador	2012-NR	Women >17y	Refugees	Not reported	Psychotherapy	Trauma-related disorder	NGO	Not reported	Local interdisciplinary team
Constandinides 2011	RCT	Palestine	2003-2010	Adolescents	Not reported	Not reported	School-based interventions, training	GMHF	Education, NGO	Schools	NGO staff, teachers
Corbin 2009	Non-Research	Uganda	2006-NR	General	IDPs	Camp	Training	GMHF	Research	Camps	Research staff, physicians, social workers
Cox 2007	Qualitative Study	B&H	1997-2001	Adolescents	Not reported	Not reported	Group therapy, psychoeducation, life skills/problem solving training, training	Trauma-related disorder, grief	Education, NGO	Schools	Counsellors, psychologists, NGO staff
Dahl 1998	Observational Study	B&H	1993-NR	Women >15y	IDPs	Not reported	Psychosocial support, counselling, educational and recreational activities	Trauma-related disorder	NGO	Specialized centres	Not reported
de Jong 2000	Observational Study	Tanzania	NR	General	Refugees	Camp	Psychosocial support	GMHF	NGO	Camps	CHWs, physicians, nurses, NGO staff
Diab 2014	RCT	Palestine	2009-2009	Adolescents	IDPs, refugees	Camp, rural, urban	CBT, mind-body techniques, psychoeducation	PTSD	Education	Schools	Counsellors, NGO staff
Donev 2002	Non-Research	Macedonia	1999-NR	General	Refugees	Camp	Psychosocial support	GMHF	NGO	Camps	Not reported
Dybdahl 2000	Observational Study	B&H	1994-1995	Women >16y	IDPs	Rural, urban	Group therapy, psychoeducation	Trauma-related disorder	NGO	Specialized centres	Psychologists, therapists
Dybdahl 2001	RCT	B&H	1996-1996	Children, caregivers	IDPs	Camp, rural	Group therapy, training	GMHF	NGO, research	Specialized centres	Teachers, psychiatrist, trained local professionals

Study ID	Report Type	Country	Duration of Intervention	Population	Displacement Type	Setting	Intervention(s)	Condition(s)	Delivery Characteristics		
									Platform	Site	Personnel
Eiling 2014	Observational Study	South Sudan	2012-2012	Children, adolescents	Not reported	Not reported	Creative arts therapy, life skills/problem solving training, training	GMHF	Education, NGO	Schools	Facilitators
Elhabiby 2015	Observational Study	Sudan	NR	General	IDPs	Camp	Psychosocial support, training, medication	GMHF	NGO	Camps	Psychiatrists
Ertl 2011	RCT	Uganda	2007-2009	Adolescents	IDPs	Camp	Narrative exposure therapy, psychoeducation, counselling	PTSD	Research	Camps	Counsellors, psychologists
Espie 2009	Observational Study	Palestine	2005-2008	General	Not reported	Not reported	Psychosocial support, group interpersonal psychotherapy, medication, screening	GMHF, psychiatric disorder	Healthcare, NGO	Specialized centres	Psychiatrists, psychologists
Firdosi 2016	Observational Study	India	2006-2007	General >15y	Not reported	Not reported	Screening	PTSD	Healthcare	Hospitals	Not reported
Frljak 1997	Observational Study	B&H	1993-1994	Women	Refugees, not displaced	Rural, urban	Psychosocial support	Trauma-related disorder	NGO	Clinics	Not reported
Gaboulaud 2010	Observational Study	Palestine	2000-2006	General	Not reported	Not reported	Psychotherapy, medication	GMHF, PTSD	NGO	Clinics, home	Therapist, psychologists, psychiatrists
Gerber 2014	Observational Study	Kosovo	1998-NR	Children, adolescents	IDPs	Camp	Creative arts therapy	GMHF	Education, NGO	Schools	Trained volunteers
Gordon 2008	RCT	Kosovo	2004-2005	Adolescents	Not reported	Not reported	Mind-body techniques	PTSD	Education, research	Schools	Teachers
Gordon 2004	Observational Study	Kosovo	1999-2000	Adolescents	Not reported	Not reported	Mind-body techniques	PTSD	Education, research	Schools	Teachers
Gupta 2008	Observational Study	Sierra Leone	1999-2000	Children, adolescents	IDPs	Camp	Group therapy, creative arts therapy, play therapy, training	Trauma-related disorder	Education, NGO	Camps	Teachers
Harrison 2013	Non-Research	Syria	2012-NR	General	IDPs, refugees	Not reported	Psychosocial support, sensitization campaigns/community mobilization	GMHF	Healthcare, NGO	Clinics, home	Volunteers

Kamali M, *et al. BMJ Global Health* 2020; 5:e002014. doi: 10.1136/bmjgh-2019-002014

Study ID	Report Type	Country	Duration of Intervention	Population	Displacement Type	Setting	Intervention(s)	Condition(s)	Delivery Characteristics		
									Platform	Site	Personnel
Hasanovic 2006	Non-Research	B&H	1999-NR	General	Not reported	Not reported	Creative arts therapy	GMHF	Healthcare, research	Hospitals	Psychiatrists, psychologists, nurses, social workers, theologians
Hashemi 2017	Observational Study	Palestine	2014-2014	Children, adolescents	IDPs	Not reported	Screening, training	GMHF	NGO	Electronic/print	Psychiatrists, trained field workers
Hemat 2017	Observational Study	Afghanistan	2014-2015	General	Not reported	Not reported	Counselling	GMHF	NGO	Clinics, hospitals	Not reported
Hijazi 2011	Non-Research	Lebanon	2007-2010	General	IDPs, refugees, hosts	Rural, urban	Training	GMHF	Healthcare, NGO	Clinics	NGO staff, psychiatrists, psychologists
Hrnjica 2000	Observational Study	Serbia	1999-2000	Children, adolescents, caregivers	Not displaced	Not reported	Play therapy, creative arts therapy, counselling	Generalized fear	Research	Not reported	Psychiatrists, psychologists, moderators
Humayun 2016	Observational Study	Pakistan	NR	General	IDPs	Camp	Counselling, CBT, psychoeducation, psychosocial support, medication, stress management	GMHF	Healthcare, NGO, research	Hospitals	CHWs, physicians, nurses, NGO and research staff, psychiatrists, psychologists, other MH professionals
Hustache 2009	Observational Study	Congo	2002-2003	Women	Not reported	Not reported	Psychosocial support	GMHF	Healthcare, NGO	Home, hospitals	Psychologists, social workers
Jan 2017	Observational Study	India	NR	General (14>35y)	Not reported	Not reported	Screening	Psychiatric disorder	Healthcare	Hospitals	Not reported
Jandric 2001	Observational Study	B&H	1992-1995	Children, adolescents	Not displaced	Not reported	Psychosocial support	GMHF	Healthcare	Hospitals	Not reported
Jefee-Bahloul 2014	Non-Research	Jordan	2013-2013	General	Refugees	Not reported	Training	GMHF	Healthcare, NGO	Electronic/print	Psychiatrists
Jones 2003	Non-Research	Kosovo	NR	Children, adolescents	IDPs	Not reported	Psychosocial support, training	GMHF	Healthcare, NGO	Clinics	Health workers, physicians, nurses, NGO staff, psychiatrists

Study ID	Report Type	Country	Duration of Intervention	Population	Displacement Type	Setting	Intervention(s)	Condition(s)	Delivery Characteristics		
									Platform	Site	Personnel
Jordans 2013	RCT	Burundi	NR	Adolescents, caregivers	IDPs	Not reported	Psychoeducation, training	Distress	Education, research	Schools	Counsellors, NGO staff
Jordans 2010	RCT	Nepal	2006-2007	Adolescents	Not reported	Not reported	Creative arts therapy, psychoeducation, narrative exposure therapy, group therapy	Distress	Education, NGO, research	Schools	Counsellors, trained local research assistants
Karam 2008	Quasi-experimental/NRCT	Lebanon	NR	Children, adolescents	Not reported	Not reported	CBT, training	Anxiety, Depression, PTSD	NGO, research	Schools	Teachers
Khan 2017	RCT	Pakistan	2012-2013	Pregnant women	IDPs	Camp, dispersed	Psychoeducation, training	GMHF	Healthcare, research	Home	CHWs
Kohrt 2011	Observational Study	Nepal	2007-2007	Adolescents	Not reported	Not reported	Screening	GMHF	NGO	Schools	Counsellors, NGO staff
Lai 2014	Non-Research	Thailand	NR	General	Refugees	Camp	Drug and alcohol rehabilitation programme	Substance addiction or misuse	NGO	Camps	Not reported
Lama 2016	Observational Study	Lebanon	2009-2013	General	Refugees	Not reported	Electroconvulsive therapy	Psychiatric disorder	Healthcare	Hospitals	Not reported
Layne 2001	Observational Study	B&H	1999-2000	Adolescents	Not reported	Not reported	Psychoeducation, group interpersonal psychotherapy, creative arts therapy	Grief, PTSD, trauma-related disorder	Education, NGO, research	Schools	Counsellors
Layne 2008	RCT	B&H	2000-2001	Adolescents	Not reported	Not reported	Psychoeducation, group interpersonal psychotherapy, training	Grief, depression, PTSD, trauma	Education, NGO, research	Schools	Counsellors
Lemaire 2001	Non-Research	B&H	1993-1996	General	IDPs, refugees	Camp	Group therapy	Trauma-related disorder	NGO	Camps	Therapists
LeRoch 2010	Observational Study	Jordan, Lebanon	2008-2009	General	Refugees	Urban	Counselling, screening, psychosocial support, play therapy	GMHF	NGO	Home, not reported	Counsellors, psychologists, social workers
Liddell 2013	Observational Study	East Timor	2004-2011	General	Not reported	Not reported	Screening	Externalizing symptoms	Research	Home	Research staff, psychologists

Study ID	Report Type	Country	Duration of Intervention	Population	Displacement Type	Setting	Intervention(s)	Condition(s)	Delivery Characteristics		
									Platform	Site	Personnel
Liebling 2016	Qualitative Study	Uganda	NR	General	IDPs	Camp	Counselling	Trauma-related disorder	Healthcare, NGO	Specialized centres	Nurses, psychiatrists, psychologists, social workers
Llosa 2012	Observational Study	Palestine	2007-2011	General	Not reported	Not reported	Group therapy, psychotherapy, psychosocial support, screening	Anxiety, depression, distress, PTSD, trauma	NGO	Clinics	Psychiatrists, psychologists, other MH professionals
Lokuge 2013	Observational Study	DRC, Iraq, Palestine	2009-2012	Children, adolescents	IDPs, not displaced	Camp, dispersed	Counselling, screening	Trauma-related disorder	NGO	Hospitals	NGO staff, trained lay counsellors
Lorschiedter 2007	Non-Research	Uganda	1997-NR	General	Not reported	Not reported	Counselling, sensitization campaigns/community mobilization	Trauma-related disorder	Not reported	Home	Community volunteer counsellors
Loughry 2006	Quasi-experimental/NRCT	Palestine	2003-2004	Children, adolescents	Not reported	Not reported	Creative arts therapy, group therapy, training	GMHF	NGO	Community centres	NGO staff, trained local volunteers
Mankuta 2012	Observational Study	DRC	2008-2008	Women	Not reported	Not reported	Screening, EMDR	Trauma-related disorder	NGO	Health posts	Physicians, nurses, psychologists, psychiatrists
McKell 2017	Qualitative Study	Jordan	NR	General	Refugees	Camp	Medication, Screening	GMHF	NGO	Camps	Physicians
Mercer 2005	Qualitative Study	India	1995-NR	General	Refugees	Not reported	Psychosocial support, training	PTSD	Healthcare, NGO	Clinics	Physicians, psychologists, health workers
Mirghani 2013	Non-Research	Syria	2007-NR	General	Refugees	Rural	Psychosocial support, training	GMHF	NGO	Not reported	NGO staff, psychologists
Mogga 2017	Non-Research	Uganda	2016-NR	Women	Refugees	Camp	CBT, psychological first aid	Anxiety, PTSD	NGO	Not reported	Psychiatrists, psychologists, social workers
Mooren 2003	Observational Study	B&H	1994-1999	General (>7y)	Not reported	Not reported	Psychotherapy	Trauma-related disorder	NGO	Specialized centres	Nurses, psychiatrists, psychologists
Morof 2014	Observational Study	Uganda	2010-2010	Women	Refugees	Urban	Counselling	PTSD	NGO	Not reported	Mental health professionals

Kamali M, *et al. BMJ Global Health* 2020; 5:e002014. doi: 10.1136/bmjgh-2019-002014

Study ID	Report Type	Country	Duration of Intervention	Population	Displacement Type	Setting	Intervention(s)	Condition(s)	Delivery Characteristics		
									Platform	Site	Personnel
Morris 2012	Quasi-experimental/NRCT	Uganda	2007-2008	Caregivers	IDPs	Camp	Psychoeducation, training	GMHF	NGO	Home	Facilitators, nutrition support workers
Muller 2011	Observational Study	Philippines	2008-2009	General	IDPs	Not reported	Psychological first aid	GMHF	Healthcare, NGO	Mobile clinics	CHWs, psychologists
Nakimuli-Mpungu 2013	Observational Study	Uganda	2005-2011	General	IDPs	Camp, dispersed	Psychotherapy, medication	Bipolar, PTSD, depression, schizophrenia	Healthcare, NGO	Clinics	Counsellors, nurses, psychiatrists, psychologists, social workers
Neuner 2002	Non-Research	Macedonia	1999-NR	General	Refugees	Camp	Psychosocial support, narrative exposure therapy	GMHF	NGO, research	Clinics	Physicians, psychologists, psychiatrists, trained refugees
Neuner 2004	RCT	Uganda	2000-2001	General	Refugees	Dispersed	Counselling, narrative exposure therapy, psychoeducation	PTSD	NGO, research	Not reported	Therapists
Neuner 2008	RCT	Uganda	2003-2004	General	Refugees	Camp	Counselling, narrative exposure therapy, training	PTSD	NGO, research	Not reported	Trained lay refugee counsellors
Newnham 2015	Observational Study	Sierra Leone	2002-NR	Adolescents, young adults (<24y)	Not reported	Not reported	CBT, training	GMHF	Research	Community centres	CHWs
Ntakarutimana 2008	Non-Research	Burundi	2005-2006	General	Not reported	Not reported	Training, psychosocial support, narrative exposue therapy	Trauma-related disorder	Faith-based, NGO	Specialized centres, schools	Facilitators, NGO staff, trainers
O'Brien 2009	Non-Research	Congo	2005-2008	General	Not reported	Not reported	Psychotherapy	PTSD	Healthcare, NGO	Clinics	Counsellors
O'Callaghan 2015	RCT	DRC	2011-2012	Children, adolescents	Not reported	Not reported	CBT, psychosocial support	Trauma-related disorder, GMHF	NGO, research	Not reported	NGO staff, social workers, teachers, trained facilitators
O'Callaghan 2014	RCT	DRC	NR	Children, adolescents	IDPs, not displaced	Rural	Psychosocial support, narrative exposure therapy, mind-body techniques	PTSD	NGO, research	Place of worship	Local lay facilitators

Study ID	Report Type	Country	Duration of Intervention	Population	Displacement Type	Setting	Intervention(s)	Condition(s)	Delivery Characteristics		
									Platform	Site	Personnel
Okello 2007	Observational Study	Uganda	2004-2004	Adolescents	IDPs	Camp	Counselling	GMHF	Research	Not reported	Research staff
Onyut 2004	Non-Research	Uganda	2003-NR	General	Refugees	Camp	Narrative exposure therapy, counselling, training	PTSD	NGO	Specialized centres	Therapists, trained refugees, other MH professionals
Ovuga 2008	Observational Study	Uganda	2006-NR	Children, adolescents	Not reported	Not reported	Psychosocial support	GMHF	Education	Schools	Not reported
Panter-Brick 2018	Quasi-experimental/NRCT	Jordan	2014-2016	Adolescents	Hosts, refugees	Not reported	Psychosocial support, training	GMHF	NGO	Specialized centres	NGO staff, trained lay volunteers
Peltonen 2012	Observational Study	Palestine	2005-2006	Adolescents	Not reported	Not reported	School-based intervention	GMHF	Education, NGO	Schools	Teachers, trained student mental health professionals
Qouta 2012	RCT	Palestine	2009-2009	Adolescents	IDPs, refugees	Camp, dispersed	CBT, mind-body techniques, psychoeducation	PTSD	Education, research	Schools	Counsellors, research staff
Quosh 2013	Non-Research	Syria	2008-NR	General	Refugees	Urban	Psychosocial support	GMHF	NGO	Specialized centres	Outreach volunteers
Rabaia 2010	Non-Research	Palestine	2002-NR	General	Not reported	Not reported	Psychosocial support	GMHF	Research	Not reported	CHWs
Rasmussen 2010	Observational Study	Chad	NR	General	Refugees	Camp	Psychosocial support	GMHF	NGO, research	Not reported	Not reported
Rees 2014	Observational Study	Uganda	NR	Adults	Refugees	Not reported	Transcendental meditation, training	PTSD	Not reported	Community centres	Facilitators
Reiffers 2013	Non-Research	Nepal	NR	General	Refugees	Camp	Psychoeducation, psychosocial support, screening, training	GMHF	NGO	Home, specialized centres	Counsellors
Roberts 2008	Observational Study	Uganda	2006-2006	General	IDPs	Camp	Screening	Trauma-related disorder	Research	Not reported	Nurses, psychiatrists
Roberts 2017	Observational Study	Ukraine	2016-2016	Adults	IDPs	Dispersed	Medication, psychosocial support, psychotherapy	GMHF	Healthcare, NGO, research	Clinics, hospitals	Physicians, psychologists, psychiatrists
Salah 2013	Observational Study	Sudan	2008-2008	Adults	IDPs	Camp	Screening	GMHF	NGO, research	Home	NGO/research staff, psychologists

Kamali M, *et al. BMJ Global Health* 2020; 5:e002014. doi: 10.1136/bmjgh-2019-002014

Study ID	Report Type	Country	Duration of Intervention	Population	Displacement Type	Setting	Intervention(s)	Condition(s)	Delivery Characteristics		
									Platform	Site	Personnel
Salem-Pickartz 2007	Non-Research	Jordan	2004-2005	General	Refugees	Camp	Training	GMHF	NGO, research	Not reported	NGO/research staff
Schei 1999	Non-Research	B&H	1993-NR	Women	Refugees	Rural, urban	Psychotherapy, psychoeducation, training	Trauma-related disorder	NGO	Specialized centres	Health workers, social workers, teachers, psychiatrists, psychologists, coordinators
Scholte 2004	Non-Research	DRC, Tanzania	1994-1995	General	Refugees	Camp	Psychoeducation, psychosocial support, creative arts therapy, life skills/problem solving training, training	GMHF	NGO	Specialized centres, camps	CHWs, NGO staff, nurses, trained refugees, psychologists, anthropologists
Sehovic 2000	Quasi-experimental/NRCT	B&H	1995-1998	Children, adolescents	IDPs	Not reported	CBT	PTSD	Education, research	Not reported	Teachers, therapists, parents
Shah 2014	Observational Study	Pakistan	2012-2012	General	Not reported	Not reported	Screening, medication	GMHF	Healthcare, NGO	Clinics, hospitals	Physicians, psychiatrists, psychologists
Shaheen 2016	Observational Study	Palestine	2007-2007	Adolescents	Not reported	Not reported	School-based intervention, training	PTSD	Education, research	Schools	Teachers, mental health professionals
Sheikh 2014	Observational Study	Nigeria	2013-2013	Adults	IDPs	Camp	Screening	Trauma-related disorder	Not reported	Not reported	Nurses, psychiatrists, psychologists
Sliep 2004	Non-Research	Uganda	2003-NR	General	Refugees	Camp	Creative arts therapy	GMHF	NGO	Not reported	Counsellors, coordinators, NGO staff, other MH professionals
Sonderegger 2011	Quasi-experimental/NRCT	Uganda	2006-NR	General (>15y)	IDPs	Camp	CBT, training	Trauma-related disorder	Research	Camps	Psychologists, trained facilitators
Stuvland 2000	Observational Study	B&H	1993-2000	General	Not reported	Not reported	Training, psychosocial support	Trauma-related disorder	Education, NGO	Schools	Teachers, NGO staff, psychologists
Szilard 2002	Observational Study	Macedonia	1999-NR	General	Refugees	Camp, dispersed	Screening	GMHF	NGO	Mobile clinics	NGO staff

Kamali M, *et al. BMJ Global Health* 2020; 5:e002014. doi: 10.1136/bmjgh-2019-002014

Study ID	Report Type	Country	Duration of Intervention	Population	Displacement Type	Setting	Intervention(s)	Condition(s)	Delivery Characteristics		
									Platform	Site	Personnel
Tanaka 2013	Observational Study	Thailand	1998-NR	Children	Refugees	Camp	School-based intervention	GMHF	NGO	Schools	Teachers
Tayler-Smith 2012	Observational Study	Liberia	2003-NR	Pregnant women	Not displaced	Not reported	Psychosocial support	Trauma-related disorder	Healthcare, NGO	Clinics	NGO staff
Thabet 2005	Quasi-experimental/NRCT	Palestine	NR	Children, adolescents	Refugees	Camp	Group therapy, psychoeducation, screening	PTSD	NGO	Schools	Teachers, physicians, psychiatrists, psychologists, social workers
Tol 2008	RCT	Indonesia	2006-2006	Children, adolescents	IDPs, not displaced	Urban	CBT, creative arts therapy, Screening, training	PTSD	Education, NGO, research	Schools	NGO/research staff, psychologists, trained local volunteers
Tol 2012	RCT	Sri Lanka	2007-2008	Children, adolescents	IDPs, not displaced	Urban	Screening, psychoeducation, creative arts therapy, counselling, CBT	Anxiety, Depression, PTSD	Education, research	Schools	Trained local non-specialized personnel
Tol 2009	Quasi-experimental/NRCT	Nepal	2003-2004	General (>12y)	IDPs	Not reported	Counselling, psychoeducation, training	GMHF	NGO	Clinics	Counsellors
Tol 2014	RCT	Burundi	2006-2007	Children, adolescents	IDPs, refugees, not displaced	Camp, dispersed	Screening, psychoeducation, creative arts therapy, CBT	Anxiety, Depression, PTSD	Education, research	Schools	Trained local non-specialized facilitators
Tufan 2013	Observational Study	Turkey	2005-2007	General	Refugees	Urban	Screening, training, medication, psychotherapy	Trauma-related disorder	NGO	Not reported	Trained legal counsellors
Ugurlu 2016	Observational Study	Turkey	NR	Children, adolescents	Refugees	Urban	Creative arts therapy	Anxiety, Depression, PTSD	Healthcare	Not reported	Therapists, trained refugee volunteers
Veronese 2018	Quasi-experimental/NRCT	Palestine	2014-NR	Children, adolescents	IDPs, refugees	Camp, urban	Training, counselling, narrative exposure therapy, play therapy, creative arts therapy	Trauma-related disorder	Education, NGO	Schools	Teachers, therapists, social workers
Vijayakumar 2017	RCT	India	NR	General	Refugees	Camp	Training, screening, contact and use of safety planning cards	Suicidal thoughts	Healthcare, NGO	Home, hospitals	NGO staff, trained community

Kamali M, *et al. BMJ Global Health* 2020; 5:e002014. doi: 10.1136/bmjgh-2019-002014

Study ID	Report Type	Country	Duration of Intervention	Population	Displacement Type	Setting	Intervention(s)	Condition(s)	Delivery Characteristics		
									Platform	Site	Personnel
											volunteers, psychiatrists
Wagner 2012	Observational Study	Iraq	NR	General	Not reported	Not reported	CBT, training	PTSD	NGO	Electronic/print	Psychiatrists, psychologists
Wessells 2006	Qualitative Study	Angola	1998-2001	Children, adolescents	IDPs	Rural	Psychosocial support, training, creative arts therapy, life skills/problem solving training	Trauma-related disorder	NGO	Community centres	Civic leaders, religious leaders, psychologists
Yeomans 2010	RCT	Burundi	2007-NR	General	IDPs	Camp, rural	Psychoeducation, screening, trauma healing and reconciliation workshop	PTSD	Research	Not reported	Civic leaders, religious leaders, psychologists, trained local facilitators
**Grey Literature**											
UNFPA, UNICEF 2018	Non-Research	Yemen	2016-NR	Adolescents	IDPs, not displaced	Not reported	Psychosocial support	GMHF	Informal governance/civic leadership, NGO	Not reported	Members of civil society organizations
UNFPA 2016	Non-Research	Nigeria, Pakistan	2014-NR	Women	IDPs, hosts	Camp, rural	Psychosocial support, sensitization campaigns/community mobilization, rebuilding social and community networks	GMHF, depression	Healthcare, NGO	Clinics, health posts	Counsellors, health workers
Save the Children 2016	Quasi-experimental/NRCT	Lebanon	2015-NR	General	Refugees	Camp, dispersed	Multipurpose cash assistance	Distress	NGO	Electronic/print	NGO staff
IRC 2014	RCT	Thailand	2011-2013	Children, caregivers	IDPs, refugees	Dispersed	Life Skills/Problem Solving Training	GMHF	NGO	Not reported	NGO staff, community-based facilitators
IRC 2014	Non-Research	Ethiopia	NR	Children, caregivers	Refugees	Camp	CBT, psychoeducation, life skills/problem solving training, training	Trauma-related disorder	NGO	Camps	Counsellors, NGO staff, psychologists
IRC 2016	Non-Research	Lebanon	2015-2016	Adolescents	IDPs, refugees	Camp, rural, urban	Psychosocial support	Trauma-related disorder	Healthcare, NGO	Clinics, place of worship, schools	Health workers, NGO staff

Study ID	Report Type	Country	Duration of Intervention	Population	Displacement Type	Setting	Intervention(s)	Condition(s)	Delivery Characteristics		
									Platform	Site	Personnel
IRC 2016	Non-Research	Chad	2014-2016	Women	IDPs	Rural	Psychosocial support	GMHF	NGO	Research centres	Health workers
ICRC 2013	Non-Research	Nepal	1998-NR	General	Not displaced	N/A	Psychosocial support	GMHF	NGO	Not reported	NGO staff
WRC 2014	Non-Research	Ethiopia	2010-2015	Adolescents	Refugees	Camp	Psychosocial support	GMHF	NGO	Community centres	NGO staff
WRC 2018	Non-Research	Jordan	2017-NR	General	Refugees	Camp, rural, urban	Counselling	GMHF	NGO	Community centres, home	NGO staff, field officers
MSF 2016	Non-Research	Burundi	2009-2015	Women	Not displaced	N/A	Psychosocial support	GMHF	Healthcare, NGO	Hospitals	Social workers
UNHCR 2018	Non-Research	Rwanda	2016-2016	Women	Refugees	Camp	Counselling	GMHF	NGO	Camps	NGO staff, psychologists
UNHCR 2013	Non-Research	Syria	NR	General	IDPs, refugees	Rural, urban	Psychosocial support, psychoeducation, screening, training	GMHF	NGO	Research centres, community centres	NGO staff, outreach refugee volunteers, psychologists, psychiatrists, social workers
UNHCR 2013	Non-Research	Colombia	2012-NR	General, Women	IDPs, refugees	Camp	Psychosocial support, screening	GMHF, trauma-related disorder	Faith-based, NGO	Specialized centres	Faith-based organization staff, NGO staff, psychologists, social workers, trained volunteers
UNICEF 2016	Non-Research	Palestine	2014-2015	Children, adolescents	IDPs, refugees	Dispersed	Psychosocial support	GMHF	NGO	Specialized centres	NGO staff
UNICEF 2016	Non-Research	South Sudan	2013-NR	Children, adolescents	IDPs, refugees	Camp, dispersed	Counselling	Trauma-related disorder	NGO	Specialized centres	NGO staff
UNICEF 2016	Non-Research	Pakistan	2011-NR	Women	IDPs, refugees	Camp	Psychosocial support	Trauma-related disorder	NGO	Not reported	Psychologists
UNICEF 2016	Non-Research	Lebanon	2015-NR	Children, adolescents, women	Hosts, refugees	Not reported	Psychosocial support	Trauma-related disorder	Faith-based, NGO	Clinics, hospitals, place of worship, schools, specialized centres	NGO staff

Study ID	Report Type	Country	Duration of Intervention	Population	Displacement Type	Setting	Intervention(s)	Condition(s)	Delivery Characteristics		
									Platform	Site	Personnel
UNICEF 2013	Non-Research	Pakistan	2011-NR	Children, adolescents, women	IDPs, hosts	Camp, rural	Psychosocial support	Trauma-related disorder	NGO	Camps, clinics, health posts, home, mobile clnics, place of worship, research centres, specialized centres	Counsellors, NGO staff, volunteers
UNICEF 2016	Non-Research	Somalia	2011-NR	Adolescents, women	IDPs, not displaced	Camp, dispersed	Psychosocial support	Trauma-related disorder	NGO	Specialized centres	NGO staff, trained community-based volunteers
UNICEF 2016	Non-Research	CAR	2014-NR	Children, adolescents, women	IDPs, not displaced	Camp, rural, urban	Psychosocial support	Trauma-related disorder	NGO	Home, specialized centres	NGO staff
UNICEF 2013	Non-Research	Congo (DR)	NR	Children, general	IDPs	Camp, dispersed	Psychosocial support, counselling	GMHF	NGO	Camps, health posts, home, specialized centres	Counsellors, NGO staff
World Vision 2013	Non-Research	Ethiopia	2012-NR	Children, adolescents	Refugees	Camp	Counselling, creative arts therapy, play therapy, psychosocial support	GMHF	NGO	Specialized centres	NGO staff
World Vision 2013	Non-Research	Uganda	2012-NR	Children, adolescents	Refugees	Camp	Creative arts therapy, group interpersonal therapy, play therapy	GMHF	NGO	Camps	NGO staff
World Vision 2017	Non-Research	Uganda	2013-2016	Children, adolescents	Refugees	Camp	Play therapy, screening, training	GMHF	NGO	Clinics	NGO staff, trained refugees

*CBT= cognitive behavioural therapy, EMDR= eye movement desensitization and reprocessing, screening= screening for referral, psychoeducation= psychoeducation therapy, GMHF= General Mental Health and functioning, NRCT= Non-randomized control trial, B&H= Bosnia and Herzegovina, Pop'n= population, other MH professionals= other mental health professionals, CHWs= community health workers, CAR= Central African Republic, DRC= Democratic Republic of Congo

Kamali M, *et al. BMJ Global Health* 2020; 5:e002014. doi: 10.1136/bmjgh-2019-002014

Appendix C: Reported quantitative data on intervention coverage and effectiveness, by outcome

Study ID	Target age (in years)	Intervention Start Year	Report Type	Intervention(s)	Description of outcome	n	Estimate (95% CI)	SD	SE	p-value	Displacement Type	Setting	Site	Personnel
**Anxiety- related Symptoms**														
Karam 2008	6-18	NR	Quasi-experimental/NRCT	CBT	Generalized estimating equation analysis for treatment effect-Group (treatment vs. control)	194	-0.44		0.41	0.284	Not reported	Not reported	Schools	Teachers
Tol 2008	7-15	2006	RCT	CBT, creative arts therapy	Mean change difference in anxiety scores between intervention and waitlist control	NR	-0.1 (-0.3, 0.6)		0.22		IDPS, Not displaced	Dispersed	Schools	Trained volunteers
Ugurlu 2016	7-12	NR	Observational study	Creative arts therapy	Mean trait anxiety scores before intervention (baseline)	63	36.92	6.96		0.05	Refugees	Dispersed	Not reported	Therapists, art therapists
Ugurlu 2016	7-12	NR	Observational study	Creative arts therapy	Mean trait anxiety scores after intervention (endline)	63	30.28	7.39		0.05	Refugees	Dispersed	Not reported	Therapists, art therapists
Dybdahl 2001	5-6	1996	RCT	Group therapy	Difference between pretest and post-test scores in control group	45	-0.11				IDPs	Camp, Dispersed	Specialized centres	Teachers, psychiatrists, local professionals
Dybdahl 2001	5-6	1996	RCT	Group therapy	Difference between pretest and post-test scores in intervention group	42	-0.41				IDPs	Camp, Dispersed	Specialized centres	Teachers, psychiatrists, local professionals
Tol 2012	9-12	2007	RCT	psychoeducation, creative arts therapy	Mean change in anxiety symptoms score among boys in treatment group (baseline vs endline)	122	1.7	2.46			IDPS, Not displaced	Dispersed	Schools	Trained volunteers
Tol 2012	9-12	2007	RCT	psychoeducation, creative arts therapy	Mean change in anxiety symptoms score among boys in waitlist control group (baseline vs endline)	123	1.07	2.21			IDPS, Not displaced	Dispersed	Schools	Trained volunteers
Tol 2012	9-12	2007	RCT	psychoeducation, creative arts therapy	Mean change in anxiety symptoms score among girls in treatment group (baseline vs endline)	76	1.29	2.44			IDPS, Not displaced	Dispersed	Schools	Trained volunteers
Tol 2012	9-12	2007	RCT	psychoeducation, creative arts therapy	Mean change in anxiety symptoms score among girls in waitlist control group (baseline vs endline)	78	1.55	2.43			IDPS, Not displaced	Dispersed	Schools	Trained volunteers
**Depression- related Symptoms**														
Karam 2008	6-18	1900	Quasi-experimental/NRCT	CBT	Generalized estimating equation analysis for treatment effect-Group (treatment vs. control)	194	-0.25		0.37	0.501	Not reported	Not reported	Schools	Teachers
Tol 2008	7-15	2006	RCT	CBT, creative arts therapy	Mean change difference in depression scores between intervention and waitlist control	NR	-0.7 (-0.1, 1.5)		0.4		IDPS, Not displaced	Dispersed	Schools	Trained volunteers
Ugurlu 2016	7-12	NR	Observational study	Creative arts therapy	Mean depression scores before intervention (baseline)	63	9.97	1.01		0.05	Refugees	Dispersed	Not reported	Therapists, art therapists
Ugurlu 2016	7-12	NR	Observational study	Creative arts therapy	Mean depression scores after intervention (endline)	63	6	4.54		0.05	Refugees	Dispersed	Not reported	Therapists, art therapists

Study ID	Target age (in years)	Intervention Start Year	Report Type	Intervention(s)	Description of outcome	n	Estimate (95% CI)	SD	SE	p-value	Displacement Type	Setting	Site	Personnel
Betancourt 2012	14-17	NR	RCT	Group therapy	Effect size estimates of APAI depression scores of group interpersonal therapy compared to wait-list control condition in nonabducted female subjects	41	1.06				IDPs	Camp	Camp	NGO and Research Staff
Betancourt 2012	14-17	NR	RCT	Group therapy	Effect size estimates of APAI depression scores of group interpersonal therapy compared to wait-list control condition in nonabducted male subjects	21	-0.02				IDPs	Camp	Camp	NGO and Research Staff
Betancourt 2012	14-17	NR	RCT	Group therapy	Effect size estimates of APAI depression scores of group interpersonal therapy compared to wait-list control condition in abducted female subjects	17	0.5				IDPs	Camp	Camp	NGO and Research Staff
Betancourt 2012	14-17	NR	RCT	Group therapy	Effect size estimates of APAI depression scores of group interpersonal therapy compared to wait-list control condition in abducted male subjects	24	0.92				IDPs	Camp	Camp	NGO and Research Staff
Dybdahl 2001	5-6	1996	RCT	Group therapy	Difference between pretest and post-test scores in control group	45	0.5				IDPs	Camp, Dispersed	Specialized centres	Teachers, psychiatrists, local professionals
Dybdahl 2001	5-6	1996	RCT	Group therapy	Difference between pretest and post-test scores in intervention group	42	-0.3				IDPs	Camp, Dispersed	Specialized centres	Teachers, psychiatrists, local professionals
Tol 2014	8-17	2006	RCT	psychoeducation, creative arts therapy	Mean change in depressive symptoms score among boys in intervention group (baseline vs endline)	77	1.42	4.61			IDPS, Not displaced	Camp, Dispersed	Schools	Trained volunteers
Tol 2014	8-17	2006	RCT	psychoeducation, creative arts therapy	Mean change in depressive symptoms score among boys in waitlist control group (baseline vs endline)	94	2.58	5.6			IDPS, Not displaced	Camp, Dispersed	Schools	Trained volunteers
Tol 2014	8-17	2006	RCT	psychoeducation, creative arts therapy	Mean change in depressive symptoms score among girls in intervention group (baseline vs endline)	76	2.21	5.42			IDPS, Not displaced	Camp, Dispersed	Schools	Trained volunteers
Tol 2014	8-17	2006	RCT	psychoeducation, creative arts therapy	Mean change in depressive symptoms score among girls in waitlist control group (baseline vs endline)	82	3	6			IDPS, Not displaced	Camp, Dispersed	Schools	Trained volunteers
Tol 2012	9-12	2007	RCT	psychoeducation, creative arts therapy	Mean change in depressive symptoms score among boys in	122	1.64	4.8			IDPS, Not displaced	Dispersed	Schools	Trained volunteers

Kamali M, *et al. BMJ Global Health* 2020; 5:e002014. doi: 10.1136/bmjgh-2019-002014

Study ID	Target age (in years)	Intervention Start Year	Report Type	Intervention(s)	Description of outcome	n	Estimate (95% CI)	SD	SE	p-value	Displacement Type	Setting	Site	Personnel
					intervention group (baseline vs endline)									
Tol 2012	9-12	2007	RCT	psychoeducation, creative arts therapy	Mean change in depressive symptoms score among boys in waitlist control group (baseline vs endline)	123	1.62	4.48			IDPS, Not displaced	Dispersed	Schools	Trained volunteers
Tol 2012	9-12	2007	RCT	psychoeducation, creative arts therapy	Mean change in depressive symptoms score among girls in intervention group (baseline vs endline)	76	1.42	3.86			IDPS, Not displaced	Dispersed	Schools	Trained volunteers
Tol 2012	9-12	2007	RCT	psychoeducation, creative arts therapy	Mean change in depressive symptoms score among girls in waitlist control group (baseline vs endline)	78	2.03	4.02			IDPS, Not displaced	Dispersed	Schools	Trained volunteers
**General Mental Health and Functioning**														
Tol 2008	7-15	2006	RCT	CBT, creative arts therapy	Mean change difference in hope scores between intervention and waitlist control	NR	-2.2 (-3.5, -0.9)		0.66		IDPS, Not displaced	Dispersed	Schools	Trained volunteers
Diab 2014	10-13	2009	RCT	CBT, mind-body techniques, psychoeducation	Wellbeing score in control group at baseline	240	36.71	9.59			IDPs, Refugees	Camp, Dispersed	Schools	Research Staff, counsellors
Diab 2014	10-13	2009	RCT	CBT, mind-body techniques, psychoeducation	Wellbeing score in control group at endline	240	36.66	8.86			IDPs, Refugees	Camp, Dispersed	Schools	Research Staff, counsellors
Diab 2014	10-13	2009	RCT	CBT, mind-body techniques, psychoeducation	Wellbeing score in intervention group at baseline	242	33.95	9.52			IDPs, Refugees	Camp, Dispersed	Schools	Research Staff, counsellors
Diab 2014	10-13	2009	RCT	CBT, mind-body techniques, psychoeducation	Wellbeing score in intervention group at endline	242	34.98	9.66			IDPs, Refugees	Camp, Dispersed	Schools	Research Staff, counsellors
Lokuge 2013	15-19	2012	Observational study	Counselling	Proportion of adolescents (15-19 years) attending mental health services who received at least 10 counselling sessions*	85	38				IDPS, Not displaced	Dispersed	Not reported	NGO workers, community members
Lokuge 2013	<15	2012	Observational study	Counselling	Proportion of children (<15 years) attending mental health services who received at least 10 counselling sessions*	213	44				IDPS, Not displaced	Dispersed	Not reported	NGO workers, community members
Dybdahl 2001	5-6	1996	RCT	Group therapy	Difference between pretest and post-test scores in control group	45	0.01				IDPs	Camp, Dispersed	Specialized centres	Teachers, psychiatrists, local professionals
Dybdahl 2001	5-6	1996	RCT	Group therapy	Difference between pretest and post-test scores in intervention group	42	-0.11				IDPs	Camp, Dispersed	Specialized centres	Teachers, psychiatrists, local professionals

Kamali M, *et al. BMJ Global Health* 2020; 5:e002014. doi: 10.1136/bmjgh-2019-002014

Study ID	Target age (in years)	Intervention Start Year	Report Type	Intervention(s)	Description of outcome	n	Estimate (95% CI)	SD	SE	p-value	Displacement Type	Setting	Site	Personnel
Annan 2016	7-15	2012	RCT	Life skills/problem solving training	Diff-in-diff treatment effect of Internalizing problems score (CBCL)	0	0.06			9.99	Refugees	Dispersed	Schools, community halls	NGO and Research Staff, CHWs, skilled facilitators
Annan 2016	7-15	2012	RCT	Life skills/problem solving training	Diff-in-diff treatment effect of Attention problems score (CBCL)	0	0.03			9.99	Refugees	Dispersed	Schools, community halls	NGO and Research Staff, CHWs, skilled facilitators
Annan 2016	7-15	2012	RCT	Life skills/problem solving training	Diff-in-diff treatment effect of Externalizing problems score (CBCL)	0	0.11			0.05	Refugees	Dispersed	Schools, community halls	NGO and Research Staff, CHWs, skilled facilitators
Annan 2016	7-15	2012	RCT	Life skills/problem solving training	Diff-in-diff treatment effect of Child Psychosocial Protective Factors Scale Score	0	0.2			0.01	Refugees	Dispersed	Schools, community halls	NGO and Research Staff, CHWs, skilled facilitators
Tol 2014	8-17	2006	RCT	psychoeducation, creative arts therapy	Mean change in hope score among boys in intervention group (baseline vs endline)	77	-1.03	6.71			IDPS, Not displaced	Camp, Dispersed	Schools	Trained volunteers
Tol 2014	8-17	2006	RCT	psychoeducation, creative arts therapy	Mean change in hope score among boys in waitlist control group (baseline vs endline)	94	-0.47	6.59			IDPS, Not displaced	Camp, Dispersed	Schools	Trained volunteers
Tol 2014	8-17	2006	RCT	psychoeducation, creative arts therapy	Mean change in hope score among girls in intervention group (baseline vs endline)	76	-2.97	6.27			IDPS, Not displaced	Camp, Dispersed	Schools	Trained volunteers
Tol 2014	8-17	2006	RCT	psychoeducation, creative arts therapy	Mean change in hope score among girls in waitlist control group (baseline vs endline)	82	-1.93	6.17			IDPS, Not displaced	Camp, Dispersed	Schools	Trained volunteers
Tanaka 2013	2-5	1998	Observational study	School-based intervention	Mean psychosocial development score of children in nursery school < 1year (control)	140	2.17		0.03		Refugees	Camp	Schools	Teachers
Tanaka 2013	2-5	1998	Observational study	School-based intervention	Mean psychosocial development score of children in nursery school > 1 year (intervention)	191	2.24		0.02		Refugees	Camp	Schools	Teachers
Loughry 2006	6-17	2003	Quasi-experimental/NRCT	Creative arts therapy, group therapy	CBCL score in West Bank comparison group at baseline	50	38.18	16.64			Not reported	Not reported	Community centres	NGO workers, community members
Loughry 2006	6-17	2003	Quasi-experimental/NRCT	Creative arts therapy, group therapy	CBCL score in West Bank comparison group at endline	50	35.42	21.9		1	Not reported	Not reported	Community centres	NGO workers, community members
Loughry 2006	6-17	2003	Quasi-experimental/NRCT	Creative arts therapy, group therapy	CBCL score in Gaza comparison group at baseline	46	43.46	18.88			Not reported	Not reported	Community centres	NGO workers, community members
Loughry 2006	6-17	2003	Quasi-experimental/NRCT	Creative arts therapy, group therapy	CBCL score in Gaza comparison group at endline	46	40.26	19.44		1	Not reported	Not reported	Community centres	NGO workers, community members

Study ID	Target age (in years)	Intervention Start Year	Report Type	Intervention(s)	Description of outcome	n	Estimate (95% CI)	SD	SE	p-value	Displacement Type	Setting	Site	Personnel
Loughry 2006	6-17	2003	Quasi-experimental/NRCT	Creative arts therapy, group therapy	CBCL score in West Bank Intervention group at baseline	200	35.88	20.71			Not reported	Not reported	Community centres	NGO workers, community members
Loughry 2006	6-17	2003	Quasi-experimental/NRCT	Creative arts therapy, group therapy	CBCL score in West Bank Intervention group at endline	200	28.69	22.29		0.003	Not reported	Not reported	Community centres	NGO workers, community members
Loughry 2006	6-17	2003	Quasi-experimental/NRCT	Creative arts therapy, group therapy	CBCL score in Gaza intervention group at baseline	99	48.74	22.23			Not reported	Not reported	Community centres	NGO workers, community members
Loughry 2006	6-17	2003	Quasi-experimental/NRCT	Creative arts therapy, group therapy	CBCL score in Gaza intervention group at endline	99	35.08	16.15		0.003	Not reported	Not reported	Community centres	NGO workers, community members
Morris 2012	NR (mothers)	2007	Quasi-experimental/NRCT	Psychoeducation	Mean maternal involvement score in the control group (baseline)	105	23.88	2.69			IDPs	Camp	Home	Psychosocial facilitator, nutrition support worker
Morris 2012	NR (mothers)	2007	Quasi-experimental/NRCT	Psychoeducation	Mean maternal involvement score in the control group (endline)	105	10.56	3.98			IDPs	Camp	Home	Psychosocial facilitator, nutrition support worker
Morris 2012	NR (mothers)	2007	Quasi-experimental/NRCT	Psychoeducation	Mean maternal involvement score in the intervention group (baseline)	132	24.38	2.65			IDPs	Camp	Home	Psychosocial facilitator, nutrition support worker
Morris 2012	NR (mothers)	2007	Quasi-experimental/NRCT	Psychoeducation	Mean maternal involvement score in the intervention group (endline)	132	26.27	2.24			IDPs	Camp	Home	Psychosocial facilitator, nutrition support worker
Morris 2012	NR (mothers)	2007	Quasi-experimental/NRCT	Psychoeducation	Mean maternal sadness/worry score in the control group (baseline)	105	10.59	3.65			IDPs	Camp	Home	Psychosocial facilitator, nutrition support worker
Morris 2012	NR (mothers)	2007	Quasi-experimental/NRCT	Psychoeducation	Mean maternal sadness/worry score in the control group (endline)	105	10.56	3.98			IDPs	Camp	Home	Psychosocial facilitator, nutrition support worker
Morris 2012	NR (mothers)	2007	Quasi-experimental/NRCT	Psychoeducation	Mean maternal sadness/worry score in the intervention group (baseline)	132	10.61	3.55			IDPs	Camp	Home	Psychosocial facilitator, nutrition support worker
Morris 2012	NR (mothers)	2007	Quasi-experimental/NRCT	Psychoeducation	Mean maternal sadness/worry score in the intervention group (endline)	132	8.42	2.95			IDPs	Camp	Home	Psychosocial facilitator, nutrition support worker
Diab 2014	10-13	2009	RCT	CBT, mind-body techniques, psychoeducation	Prosociality score in control group at baseline	240	3.37	1.35			IDPs, Refugees	Camp, Dispersed	Schools	Research Staff, counsellors

Study ID	Target age (in years)	Intervention Start Year	Report Type	Intervention(s)	Description of outcome	n	Estimate (95% CI)	SD	SE	p-value	Displacement Type	Setting	Site	Personnel
Diab 2014	10-13	2009	RCT	CBT, mind-body techniques, psychoeducation	Prosociality score in control group at endline	240	3.47	1.3			IDPs, Refugees	Camp, Dispersed	Schools	Research Staff, counsellors
Diab 2014	10-13	2009	RCT	CBT, mind-body techniques, psychoeducation	Prosociality score in intervention group at baseline	242	3.23	1.39			IDPs, Refugees	Camp, Dispersed	Schools	Research Staff, counsellors
Diab 2014	10-13	2009	RCT	CBT, mind-body techniques, psychoeducation	Prosociality score in intervention group at endline	242	3.22	1.4			IDPs, Refugees	Camp, Dispersed	Schools	Research Staff, counsellors
**Post-traumatic Stress Disorder**														
Karam 2008	6-18	NR	Quasi-experimental/NRCT	CBT	GEE analysis for treatment effect-Group (treatment vs. control)	194	0.22		0.34	0.517	Not reported	Not reported	Schools	Teachers
Tol 2008	7-15	2006	RCT	CBT, creative arts therapy	Mean change difference in PTSS scores between intervention and waitlist control	NR	-2.8 (1.0, 4.5)		0.89		IDPS, Not displaced	Dispersed	Schools	Trained volunteers
Diab 2014	10-13	2009	RCT	CBT, mind-body techniques, psychoeducation	PTSS scores at baseline for girls in control group	120	26.9	10.4			IDPs, Refugees	Camp, Dispersed	Schools	Research Staff, counsellors
Diab 2014	10-13	2009	RCT	CBT, mind-body techniques, psychoeducation	PTSS scores at post intervention for girls in control group	120	28.2	12.2			IDPs, Refugees	Camp, Dispersed	Schools	Research Staff, counsellors
Diab 2014	10-13	2009	RCT	CBT, mind-body techniques, psychoeducation	PTSS scores at baseline for boys in control group	120	28.6	10.9			IDPs, Refugees	Camp, Dispersed	Schools	Research Staff, counsellors
Diab 2014	10-13	2009	RCT	CBT, mind-body techniques, psychoeducation	PTSS scores at post intervention for boys in control group	120	26.6	10.9			IDPs, Refugees	Camp, Dispersed	Schools	Research Staff, counsellors
Diab 2014	10-13	2009	RCT	CBT, mind-body techniques, psychoeducation	PTSS scores at baseline for girls in intervention group	120	33.5	8.5			IDPs, Refugees	Camp, Dispersed	Schools	Research Staff, counsellors
Diab 2014	10-13	2009	RCT	CBT, mind-body techniques, psychoeducation	PTSS scores at post intervention for girls in intervention group	120	27.3	11.7			IDPs, Refugees	Camp, Dispersed	Schools	Research Staff, counsellors
Diab 2014	10-13	2009	RCT	CBT, mind-body techniques, psychoeducation	PTSS scores at baseline for boys in intervention group	122	32.1	10.6			IDPs, Refugees	Camp, Dispersed	Schools	Research Staff, counsellors
Diab 2014	10-13	2009	RCT	CBT, mind-body techniques, psychoeducation	PTSS scores at post intervention for boys in intervention group	122	24.6	10.3			IDPs, Refugees	Camp, Dispersed	Schools	Research Staff, counsellors
Ugurlu 2016	7-12	NR	Observational study	Creative arts therapy	Mean PTSD score before intervention (baseline)	63	29.8	10.5		0.001	Refugees	Dispersed	Not reported	Therapists, art therapists
Ugurlu 2016	7-12	NR	Observational study	Creative arts therapy	Mean PTSD score after the art therapy intervention (endline)	63	15.32	9.59		0.05	Refugees	Dispersed	Not reported	Therapists, art therapists
Mankuta 2012	NR (women)	2008	Observational study	EMDR	# of women sexually traumatized and diagnosed with PTSD provided with EMDR*	52	23				Not reported	Not reported	Health posts	Doctors, nurses, psychologists
Gordon 2004	12-19	1999	Observational study	Mind-body techniques	Mean PTSD score in Kosovo High School Students in Group 1 (baseline)	41	8.3	3.9			Not reported	Not reported	Schools	Teachers

Study ID	Target age (in years)	Intervention Start Year	Report Type	Intervention(s)	Description of outcome	n	Estimate (95% CI)	SD	SE	p-value	Displacement Type	Setting	Site	Personnel
Gordon 2004	12-19	1999	Observational study	Mind-body techniques	Mean PTSD score in Kosovo High School Students in Group 2 (baseline)	47	10.8	2.4			Not reported	Not reported	Schools	Teachers
Gordon 2004	12-19	1999	Observational study	Mind-body techniques	Mean PTSD score in Kosovo High School Students in Group 3 (baseline)	51	11.4	2.4			Not reported	Not reported	Schools	Teachers
Gordon 2004	12-19	1999	Observational study	Mind-body techniques	Mean PTSD score in Kosovo High School Students in Group 1 (endline)	41	6.1	2.6			Not reported	Not reported	Schools	Teachers
Gordon 2004	12-19	1999	Observational study	Mind-body techniques	Mean PTSD score in Kosovo High School Students in Group 2 (endline)	47	5.8	2.4			Not reported	Not reported	Schools	Teachers
Gordon 2004	12-19	1999	Observational study	Mind-body techniques	Mean PTSD score in Kosovo High School Students in Group 3 (endline)	51	5.5	2.5			Not reported	Not reported	Schools	Teachers
Gordon 2008	14-18	2004	RCT	Mind-body techniques	Mean PTSD scores in control group (baseline)	40	2.4	0.4			Not reported	Not reported	Schools	Teachers
Gordon 2008	14-18	2004	RCT	Mind-body techniques	Mean PTSD scores in control group (endline)	39	2.1	0.3			Not reported	Not reported	Schools	Teachers
Gordon 2008	14-18	2004	RCT	Mind-body techniques	Mean PTSD scores in intervention group (baseline)	38	2.5	0.3			Not reported	Not reported	Schools	Teachers
Gordon 2008	14-18	2004	RCT	Mind-body techniques	Mean PTSD scores in intervention group (endline)	38	2	0.3			Not reported	Not reported	Schools	Teachers
Tol 2014	8-17	2006	RCT	psychoeducation, creative arts therapy	Mean change in PTSD score among boys in intervention group (baseline vs endline)	77	5.68	9.65			IDPS, Not displaced	Camp, Dispersed	Schools	Trained volunteers
Tol 2014	8-17	2006	RCT	psychoeducation, creative arts therapy	Mean change in PTSD score among boys in waitlist control group (baseline vs endline)	94	4.85	8.56			IDPS, Not displaced	Camp, Dispersed	Schools	Trained volunteers
Tol 2014	8-17	2006	RCT	psychoeducation, creative arts therapy	Mean change in PTSD score among girls in intervention group (baseline vs endline)	76	5.85	10.97			IDPS, Not displaced	Camp, Dispersed	Schools	Trained volunteers
Tol 2014	8-17	2006	RCT	psychoeducation, creative arts therapy	Mean change in PTSD score among girls in waitlist control group (baseline vs endline)	82	5.18	11.46			IDPS, Not displaced	Camp, Dispersed	Schools	Trained volunteers
Tol 2012	9-12	2007	RCT	psychoeducation, creative arts therapy	Mean change in PTSD score among boys in intervention group (baseline vs endline)	122	5.49	8.15			IDPS, Not displaced	Dispersed	Schools	Trained volunteers
Tol 2012	9-12	2007	RCT	psychoeducation, creative arts therapy	Mean change in PTSD score among boys in waitlist control group (baseline vs endline)	123	5.49	7.75			IDPS, Not displaced	Dispersed	Schools	Trained volunteers
Tol 2012	9-12	2007	RCT	psychoeducation, creative arts therapy	Mean change in PTSD score among girls in intervention group (baseline vs endline)	76	4.83	8.17			IDPS, Not displaced	Dispersed	Schools	Trained volunteers

Study ID	Target age (in years)	Intervention Start Year	Report Type	Intervention(s)	Description of outcome	n	Estimate (95% CI)	SD	SE	p-value	Displacement Type	Setting	Site	Personnel
Tol 2012	9-12	2007	RCT	psychoeducation, creative arts therapy	Mean change in PTSD score among girls in waitlist control group (baseline vs endline)	78	7.88	8.49			IDPS, Not displaced	Dispersed	Schools	Trained volunteers
O'Callaghan 2014	7-18	NR	RCT	Psychosocial support, narrative exposure therapy, mind-body techniques	Mean CRIES score in control group (endline)	80	12.17	5.7			IDPS, Not displaced	Dispersed	Place of worship	Local lay facilitators
O'Callaghan 2014	7-18	NR	RCT	Psychosocial support, narrative exposure therapy, mind-body techniques	Mean CRIES score in intervention group (baseline)	79	11.8 (10.6, 13.0)				IDPS, Not displaced	Dispersed	Place of worship	Local lay facilitators
O'Callaghan 2014	7-18	NR	RCT	Psychosocial support, narrative exposure therapy, mind-body techniques	Mean CRIES score in intervention group (endline)	79	10.0 (9.0, 11.1)				IDPS, Not displaced	Dispersed	Place of worship	Local lay facilitators
**Trauma-related disorder**														
Bass 2013	21-50	2010	RCT	Psychosocial support	Mean scores of depression and anxiety (baseline)	248	2.2	0.5		0.0001	Not reported	Not reported	Not reported	Psychosocial assistants
Bass 2013	21-50	2010	RCT	Psychosocial support	Mean scores of depression and anxiety (endline)	248	1.7	0.7		0.0001	Not reported	Not reported	Not reported	Psychosocial assistants
Veronese 2018	8-14	2014	Quasi-experimental/NRCT	narrative exposure therapy, play therapy, creative arts therapy	Overall life satisfaction in control group (baseline)	20	17.25	1.7			IDPs, Refugees	Camp, Dispersed	Schools	Teachers, therapists, social workers
Veronese 2018	8-14	2014	Quasi-experimental/NRCT	narrative exposure therapy, play therapy, creative arts therapy	Overall life satisfaction in control group (endline)	20	13.8	1.97			IDPs, Refugees	Camp, Dispersed	Schools	Teachers, therapists, social workers
Veronese 2018	8-14	2014	Quasi-experimental/NRCT	narrative exposure therapy, play therapy, creative arts therapy	Happiness score in control group (baseline)	28	6.11	1.31			IDPs, Refugees	Camp, Dispersed	Schools	Teachers, therapists, social workers
Veronese 2018	8-14	2014	Quasi-experimental/NRCT	narrative exposure therapy, play therapy, creative arts therapy	Happiness score in control group (endline)	25	5.2	1.65			IDPs, Refugees	Camp, Dispersed	Schools	Teachers, therapists, social workers
Veronese 2018	8-14	2014	Quasi-experimental/NRCT	narrative exposure therapy, play therapy, creative arts therapy	Pessimism score in control group (baseline)	34	8.41	3.68			IDPs, Refugees	Camp, Dispersed	Schools	Teachers, therapists, social workers

Study ID	Target age (in years)	Intervention Start Year	Report Type	Intervention(s)	Description of outcome	n	Estimate (95% CI)	SD	SE	p-value	Displacement Type	Setting	Site	Personnel
Veronese 2018	8-14	2014	Quasi-experimental/NRCT	narrative exposure therapy, play therapy, creative arts therapy	Pessimism score in control group (endline)	33	7.54	3.34			IDPs, Refugees	Camp, Dispersed	Schools	Teachers, therapists, social workers
Veronese 2018	8-14	2014	Quasi-experimental/NRCT	narrative exposure therapy, play therapy, creative arts therapy	Optimism score in control group (baseline)	34	3	2.7			IDPs, Refugees	Camp, Dispersed	Schools	Teachers, therapists, social workers
Veronese 2018	8-14	2014	Quasi-experimental/NRCT	narrative exposure therapy, play therapy, creative arts therapy	Optimism score in control group (endline)	33	6.3	3.49			IDPs, Refugees	Camp, Dispersed	Schools	Teachers, therapists, social workers
Veronese 2018	8-14	2014	Quasi-experimental/NRCT	narrative exposure therapy, play therapy, creative arts therapy	Overall life satisfaction in intervention group (baseline)	24	17.64	1.53			IDPs, Refugees	Camp, Dispersed	Schools	Teachers, therapists, social workers
Veronese 2018	8-14	2014	Quasi-experimental/NRCT	narrative exposure therapy, play therapy, creative arts therapy	Overall life satisfaction in intervention group (endline)	19	18	1.18			IDPs, Refugees	Camp, Dispersed	Schools	Teachers, therapists, social workers
Veronese 2018	8-14	2014	Quasi-experimental/NRCT	narrative exposure therapy, play therapy, creative arts therapy	Happiness score in intervention group (baseline)	28	5.96	1.99			IDPs, Refugees	Camp, Dispersed	Schools	Teachers, therapists, social workers
Veronese 2018	8-14	2014	Quasi-experimental/NRCT	narrative exposure therapy, play therapy, creative arts therapy	Happiness score in intervention group (endline)	28	6.1	1.31			IDPs, Refugees	Camp, Dispersed	Schools	Teachers, therapists, social workers
Veronese 2018	8-14	2014	Quasi-experimental/NRCT	narrative exposure therapy, play therapy, creative arts therapy	Pessimism score in intervention group (baseline)	28	8.32	4.52			IDPs, Refugees	Camp, Dispersed	Schools	Teachers, therapists, social workers
Veronese 2018	8-14	2014	Quasi-experimental/NRCT	narrative exposure therapy, play therapy, creative arts therapy	Pessimism score in intervention group (endline)	28	10.61	3.64			IDPs, Refugees	Camp, Dispersed	Schools	Teachers, therapists, social workers
Veronese 2018	8-14	2014	Quasi-experimental/NRCT	narrative exposure therapy, play therapy, creative arts therapy	Optimism score in intervention group (baseline)	28	5.29	4.19			IDPs, Refugees	Camp, Dispersed	Schools	Teachers, therapists, social workers

Kamali M, *et al. BMJ Global Health* 2020; 5:e002014. doi: 10.1136/bmjgh-2019-002014

Study ID	Target age (in years)	Intervention Start Year	Report Type	Intervention(s)	Description of outcome	n	Estimate (95% CI)	SD	SE	p-value	Displacement Type	Setting	Site	Personnel
Veronese 2018	8-14	2014	Quasi-experimental/NRCT	narrative exposure therapy, play therapy, creative arts therapy	Optimism score in intervention group (endline)	28	3.93	2.89			IDPs, Refugees	Camp, Dispersed	Schools	Teachers, therapists, social workers
Bass 2013	21-50	2010	RCT	Cognitive processing therapy	Mean scores of depression and anxiety (baseline)	157	2	0.5		0.0001	Not reported	Not reported	Not reported	Psychosocial assistants
Bass 2013	21-50	2010	RCT	Cognitive processing therapy	Mean scores of depression and anxiety (endline)	157	0.8	0.6		0.0001	Not reported	Not reported	Not reported	Psychosocial assistants

Diff-in-diff= difference-in-difference, CBCL= child behaviour checklist, n=sample size, NR= not reported

*Publications reporting on intervention coverage
